# Supplementary material for: Genetic architecture of white matter microstructure captured by unsupervised deep representation learning of fractional anisotropy maps
Source: Nat Commun. 2026 Jun 3;17:7150. doi: 10.1038/s41467-026-73996-z (PMC13396798; doi:10.1038/s41467-026-73996-z)
Supplement: Supplementary file 1 — Supplementary Information [file 41467_2026_73996_MOESM1_ESM.pdf]

Supplementary Information

Genetic architecture of white matter microstructure captured by unsupervised deep representation learning of fractional anisotropy maps

Xingzhong Zhao[1], Ziqian Xie[1], Wei He[1], Hyun Yong Koh[1], Bohong Guo[2], Han Chen[3], Myriam Fornage[2,4], Degui Zhi[1, *]

1. McWilliams School of Biomedical Informatics, University of Texas Health Science Center, Houston, TX, 77030, USA.

2. School of Public Health, University of Texas Health Science Center, Houston, TX, 77030, USA.

3. Rory Meyers College of Nursing, New York University, New York, NY, 10010, USA

4. McGovern Medical School, University of Texas Health Science Center, Houston, TX, 77030, USA

Supplementary Figures

Supplementary Fig. 1 | Architecture of the UDR-WM autoencoder. Network architecture used for unsupervised deep representation modeling of white matter (UDR-WM). Whole-brain fractional anisotropy (FA) maps were encoded by a three-dimensional convolutional autoencoder into a 128-dimensional latent representation, termed unsupervised deep imaging phenotypes of FA (UDIP-FA), and then decoded to reconstruct the input FA map. WM, white matter.

Supplementary Fig. 2 | Phenotypic and genetic correlations among UDIP-FA dimensions. Pairwise association matrix for the 128 unsupervised deep imaging phenotypes of fractional anisotropy (UDIP-FA). The lower-left triangle shows phenotypic correlations estimated using Pearson correlation coefficients after covariate adjustment. The upper-right triangle shows genetic correlations estimated using linkage disequilibrium score regression (LDSC). The numbers along the rows and columns denote UDIP-FA dimensions. All correlation coefficients are shown as absolute values.

Supplementary Fig. 3 | UMAP visualization of tract-specific FA variation across the UDIP-FA latent space. Two-dimensional Uniform Manifold Approximation and Projection (UMAP) embeddings of the 128-dimensional UDIP-FA representations are shown for all participants. Each point represents one individual and is colored by the individual-level mean fractional anisotropy (FA) value within the indicated white matter (WM) tract, expressed as a percentile across the cohort. Marginal histograms show sample density along UMAP1 and UMAP2. The displayed tracts include the left and right anterior corona radiata, the left fornix cres/stria terminalis, and the genu of the corpus callosum.

Supplementary Fig. 4 | Shared variance between UDIP-FA and structural or anthropometric phenotypes. Canonical correlation analysis (CCA) was used to estimate the proportion of variance in the 128 UDIP-FA features explained by different imaging-derived and anthropometric feature sets. Feature sets include tract-averaged fractional anisotropy (FA), radial diffusivity (RD), mean diffusivity (MD), cortical surface area, cortical volume, mean cortical thickness, and body mass index (BMI). UDIP-FA, unsupervised deep imaging phenotypes of fractional anisotropy.

Supplementary Fig. 5 | Tissue enrichment of UDIP-FA perturbation maps. Heatmap showing enrichment of perturbation-based spatial maps for each UDIP-FA dimension across three tissue classes. Each row corresponds to one UDIP-FA dimension, and columns correspond to cerebrospinal fluid (CSF), grey matter (GM), and white matter (WM). Color intensity indicates the normalized Kolmogorov-Smirnov (K-S) statistic, with higher values indicating stronger enrichment of the corresponding UDIP-FA map in that tissue class. UDIP-FA, unsupervised deep imaging phenotypes of fractional anisotropy.

Supplementary Fig. 6 | Spatial enrichment patterns of UDIP-FA dimensions 1 and 2. Spatial visualization of two UDIP-FA dimensions using perturbation-based decoder interpretation (PerDI). The top row shows observed voxel-wise t values for UDIP-FA dimensions 1 and 2 across white matter (WM) tract regions. The bottom row shows WM regions significantly enriched for each dimension based on the PerDI-derived t maps and permutation testing with multiple-comparison correction. UDIP-FA, unsupervised deep imaging phenotypes of fractional anisotropy.

Supplementary Fig. 7 | Prediction of age and sex using traditional IDPs and UDIP-FA features. a, Prediction performance using traditional white matter (WM) tract imaging-derived phenotypes (IDPs). The left panel shows support vector regression (SVR) age prediction under five-fold cross-validation (CV), with predicted age plotted against chronological age; the red dashed line indicates perfect prediction. The right panel shows the mean receiver operating characteristic (ROC) curve for support vector machine (SVM) sex classification. Values are mean +/- s.d. across CV folds.

Supplementary Fig. 8 | Comparative disorder-classification performance of FA-derived phenotypes. Boxplots show the distribution of area under the receiver operating characteristic curve (AUC) values across five cross-validation folds for classification of six brain disorders using models trained on all fractional anisotropy (FA)-derived phenotypes (blue) or tract-averaged WM FA measures (orange). Center lines indicate medians, boxes indicate the interquartile range (IQR), and whiskers extend to the most extreme values within 1.5 x IQR. Points denote AUC values from individual folds, and the grey dashed line

indicates chance-level performance (AUC = 0.5). PD, Parkinson's disease; AD, Alzheimer's disease; DEP, depression; MS, multiple sclerosis; EPI, epilepsy; SCZ, schizophrenia; WM, white matter.

Supplementary Fig. 9 | Classification performance for smaller-sample disorders. Area under the receiver operating characteristic curve (AUC) values are shown for classification of Parkinson's disease (PD), Alzheimer's disease (AD), and schizophrenia (SCZ) using UDIP-FA features under five-fold cross-validation. Boxplots summarize AUC distributions across folds; center lines indicate medians, boxes indicate the interquartile range (IQR), and whiskers extend to 1.5 x IQR. Overlaid points denote individual cross-validation folds. UDIP-FA, unsupervised deep imaging phenotypes of fractional anisotropy.

Supplementary Fig. 10 | Comparison of SNP heritability across FA feature representations. Bar plot showing single nucleotide polymorphism (SNP)-based heritability (h2) for three fractional anisotropy (FA) feature representations: 128 UDIP-FA latent features, the top 21 principal components (PCs) explaining 90% of the variance in white matter imaging-derived phenotypes (IDPs), and the top 905 PCs explaining 90% of the variance in whole-brain voxel-wise FA maps. Bars show point estimates with standard errors (s.e.), and numerical labels denote estimate +/- s.e. Horizontal brackets summarize pairwise comparisons using two-sided Mann-Whitney U tests. ***P < 0.001; n.s., not significant.

Supplementary Fig. 11 | Multi-stage GWAS workflow for UDIP-FA. Workflow for genome-wide association study (GWAS) analysis of UDIP-FA features. The study was divided into discovery (N = 17,376) and replication (N = 8,499) phases, each including 128 UDIP-FA dimensions. Single-variant GWAS was performed for each dimension in each phase, followed by multivariate GWAS using joint analysis of multiple phenotypes GWAS (JAGWAS). Meta-analysis was then conducted across discovery and replication single-variant results to generate 128 meta-GWAS summary statistics. Finally, multivariate meta-GWAS was performed using JAGWAS to identify joint genetic associations across UDIP-FA dimensions. UDIP-FA, unsupervised deep imaging phenotypes of fractional anisotropy.

Supplementary Fig. 12 | Manhattan plots for multivariate GWAS of UDIP-FA. Manhattan plots show multivariate genome-wide association study (mvGWAS) results for UDIP-FA. a, Discovery cohort mvGWAS. b, Replication cohort mvGWAS. c, Meta-analysis combining the discovery and replication cohorts. Each point represents a single nucleotide polymorphism (SNP) plotted by genomic position and -log10(P) value. The top 30 most significant loci are annotated. UDIP-FA, unsupervised deep imaging phenotypes of fractional anisotropy.

Supplementary Fig. 13 | Colocalized association signals for UDIP-FA and total body BMD at 7q31.31. Top, Regional association plots for UDIP-FA and total body bone mineral density (BMD) at the 7q31.31 locus, showing -log10(P) values by hg19 genomic position; lead single nucleotide polymorphisms (SNPs) are highlighted in red and recombination rate is shown in blue. Bottom left, Scatter plot comparing SNP -log10(P) values for UDIP-FA and total body BMD, colored by linkage disequilibrium (LD; r2) with rs3801387. Bottom right, zoomed plots from 120.8 to 121.2 Mb show a shared association peak near WNT16, with CPED1 and FAM3C annotated.

Supplementary Fig. 14 | Overlap of UFAGs identified by gene-mapping strategies. Venn diagram showing overlap among gene sets identified from the meta multivariate GWAS (meta-mvGWAS) of UDIP-FA using different gene-mapping strategies. Numbers indicate genes unique to, or shared across, the corresponding methods. eQTL, expression quantitative trait locus mapping; MAGMA, Multi-marker Analysis of Genomic Annotation.

Supplementary Fig. 15 | Overlap of UFAG-interacting risk genes across brain disorders. UpSet plots show overlap among disease risk genes interacting with UDIP-FA-associated genes (UFAGs) in brain-specific molecular networks. a, Brain-specific transcriptional regulatory network (TRN). b, Brain-specific co-expression network. Bar charts above each matrix indicate intersection sizes, and horizontal bars on the left indicate set sizes for individual disorders. AD, Alzheimer's disease; ADHD, attention-deficit/hyperactivity disorder; ASD, autism spectrum disorder; BIP, bipolar disorder; DEP, depression; MS, multiple sclerosis; PD, Parkinson's disease; SCZ, schizophrenia.

Supplementary Fig. 16 | Brain-specific TRN hub module linking UFAGs and disease risk genes. Network visualization of a pivotal hub module for UDIP-FA-associated genes (UFAGs) in the brain-specific transcriptional regulatory network (TRN). Diamonds represent UFAGs, circles represent brain disorder risk genes, and edges represent regulatory relationships. Pie-chart node colors indicate overlap across disorder-associated gene sets. Colors denote Alzheimer's disease (AD; mint green),

attention-deficit/hyperactivity disorder (ADHD; pale yellow), autism spectrum disorder (ASD; lavender), bipolar disorder (BIP; coral red), depression (DEP; blue), multiple sclerosis (MS; orange), Parkinson's disease (PD; pink), and schizophrenia (SCZ; light green).

Supplementary Fig. 17 | Head-to-head comparison of loci discovery between PC-FA and UDIP-FA. a, Bar plot comparing genetic discovery between principal component (PC)-based traditional fractional anisotropy (FA) phenotypes and unsupervised deep imaging phenotypes of FA (UDIP-FA). Grey bars denote PC-based FA phenotypes, and orange bars denote UDIP-FA. The categories include risk loci, lead single nucleotide polymorphisms (SNPs), independent significant SNPs, and mapped genes. Numbers above bars indicate counts for each representation.

Supplementary Fig. 18 | Head-to-head comparison of trait prediction using PC and UDIP-FA features. a, Age prediction and sex classification using principal component (PC) features derived from traditional fractional anisotropy (FA) phenotypes. The left panel shows support vector regression (SVR) age prediction under five-fold cross-validation (CV), and the right panel shows the mean receiver operating characteristic (ROC) curve for sex classification. b, Corresponding prediction results using unsupervised deep imaging phenotypes of FA (UDIP-FA). The red dashed line indicates perfect age prediction, and the diagonal dashed line in ROC plots indicates random classification. MAE, mean absolute error; AUC, area under the ROC curve; s.d., standard deviation.

Supplementary Fig. 19 | Head-to-head comparison of PC and UDIP-FA in biological enrichment and reliability. a, Comparison of predefined FA-relevant pathway enrichment between principal component (PC)-based FA phenotypes and unsupervised deep imaging phenotypes of FA (UDIP-FA). Bars show -log10(P) for the best pathway within each predefined biological module, including axon guidance, myelin, oligodendrocyte, glial development, neurofascin, synaptic signaling, and neuron projection modules. b, Reliability and biological relevance comparison based on counts of loci overlapping prior diffusion magnetic resonance imaging (dMRI), diffusion tensor imaging (DTI), or white matter genome-wide association studies (GWAS), loci beyond prior GWAS Catalog evidence, and brain regulatory mapped genes. Grey bars denote PC-based features, and orange bars denote UDIP-FA.

Supplementary Fig. 20 | Stability and model selection of UDIP-FA across latent dimensions. a, Pairwise canonical correlation analysis (CCA) heatmap showing similarity among UDIP-FA feature sets extracted from models with different latent dimensions (32, 64, 128, 256, 512, and 1024). High CCA correlations indicate stable latent structure across model sizes. b, Sensitivity analysis of genome-wide significant single nucleotide polymorphism (SNP) discovery across latent dimensions after Bonferroni correction. The 128-dimensional UDIP-FA model selected for the main analysis provides robust discovery power while maintaining model parsimony. UDIP-FA, unsupervised deep imaging phenotypes of fractional anisotropy.

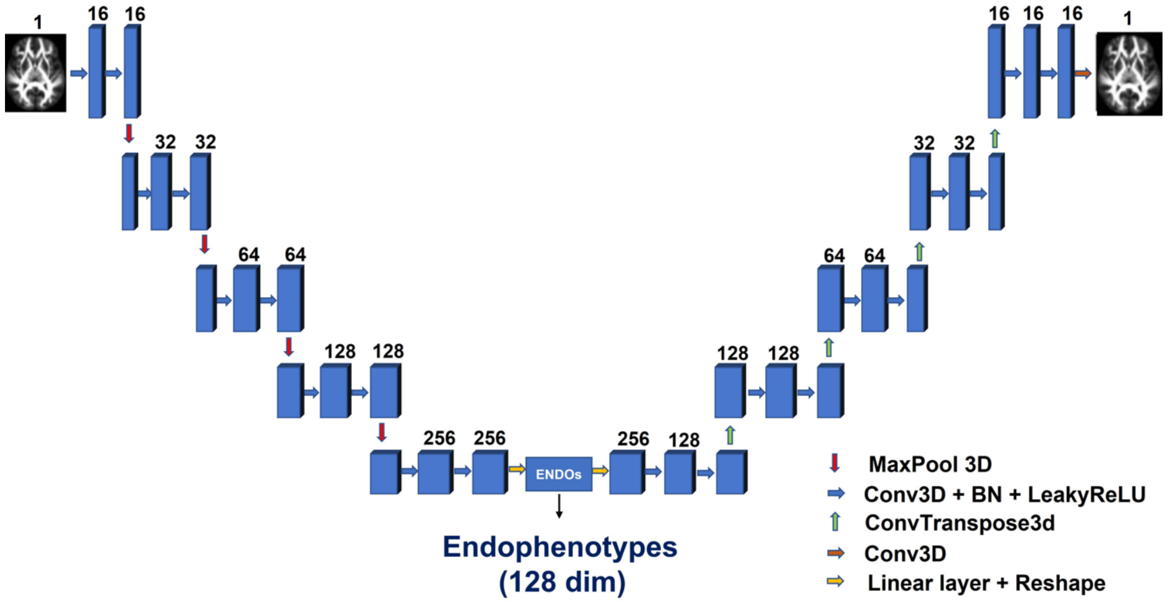

Supplementary Fig. 1 | Architecture of the UDR-WM autoencoder. Network architecture used for unsupervised deep representation modeling of white matter (UDR-WM). Whole-brain fractional anisotropy (FA) maps were encoded by a three-dimensional convolutional autoencoder into a 128-dimensional latent representation, termed unsupervised deep imaging phenotypes of FA (UDIP-FA), and then decoded to reconstruct the input FA map. WM, white matter.

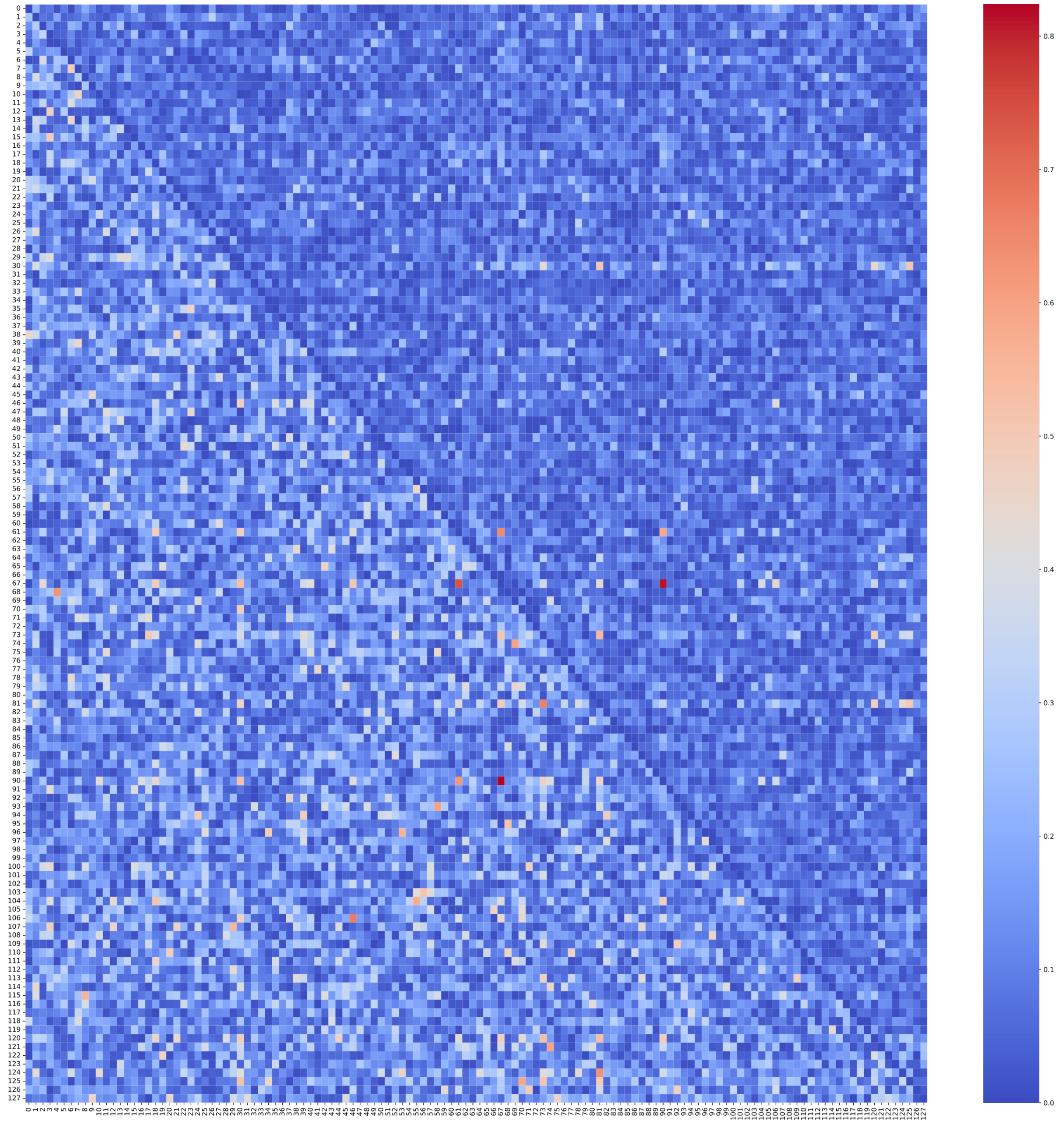

Supplementary Fig. 2 | Phenotypic and genetic correlations among UDIP-FA dimensions. Pairwise association matrix for the 128 unsupervised deep imaging phenotypes of fractional anisotropy (UDIP-FA). The lower-left triangle shows phenotypic correlations estimated using Pearson correlation coefficients after covariate adjustment. The upper-right triangle shows genetic correlations estimated using linkage disequilibrium score regression (LDSC). The numbers along the rows and columns denote UDIP-FA dimensions. All correlation coefficients are shown as absolute values.

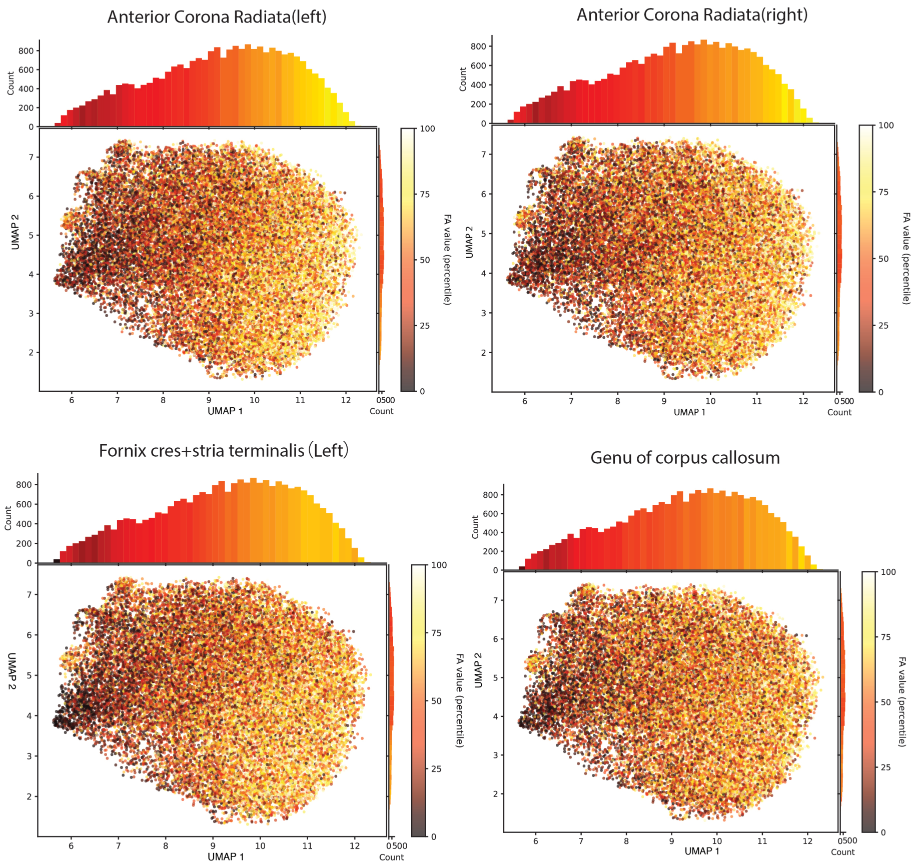

Supplementary Fig. 3 | UMAP visualization of tract-specific FA variation across the UDIP-FA latent space. Two-dimensional Uniform Manifold Approximation and Projection (UMAP) embeddings of the 128-dimensional UDIP-FA representations are shown for all participants. Each point represents one individual and is colored by the individual-level mean fractional anisotropy (FA) value within the indicated white matter (WM) tract, expressed as a percentile across the cohort. Marginal histograms show sample density along UMAP1 and UMAP2. The displayed tracts include the left and right anterior corona radiata, the left fornix cres/stria terminalis, and the genu of the corpus callosum.

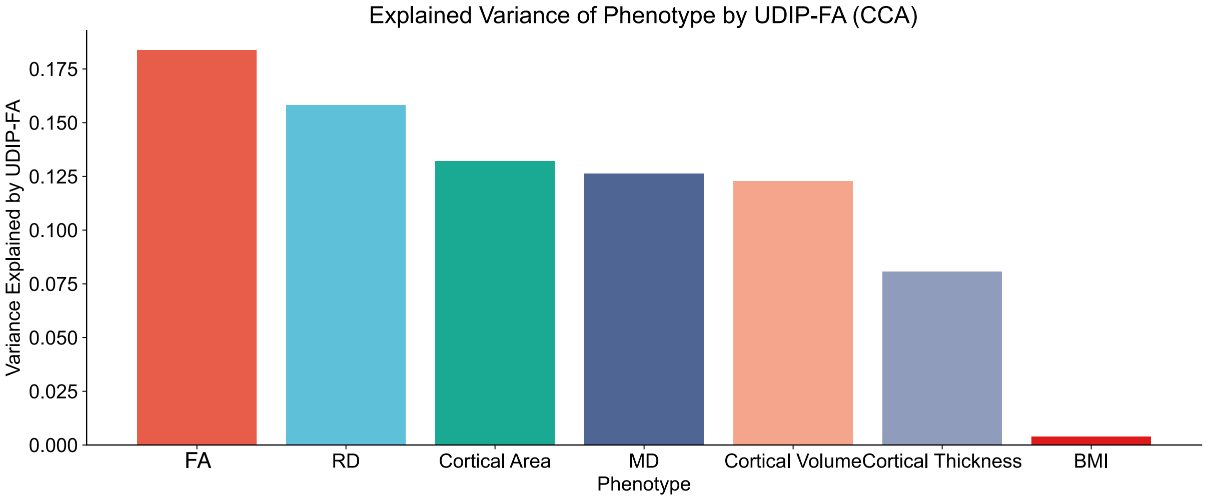

Supplementary Fig. 4 | Shared variance between UDIP-FA and structural or anthropometric phenotypes. Canonical correlation analysis (CCA) was used to estimate the proportion of variance in the 128 UDIP-FA features explained by different imaging-derived and anthropometric feature sets. Feature sets include tract-averaged fractional anisotropy (FA), radial diffusivity (RD), mean diffusivity (MD), cortical surface area, cortical volume, mean cortical thickness, and body mass index (BMI). UDIP-FA, unsupervised deep imaging phenotypes of fractional anisotropy.

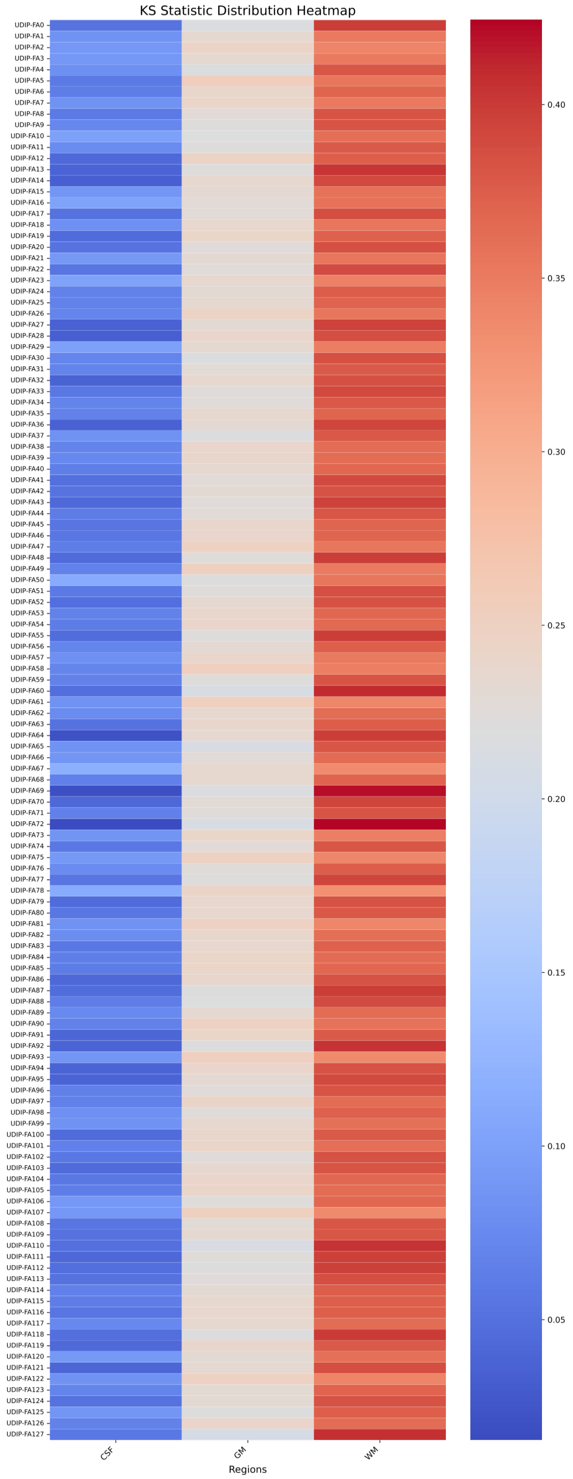

Supplementary Fig. 5 | Tissue enrichment of UDIP-FA perturbation maps. Heatmap showing enrichment of perturbation-based spatial maps for each UDIP-FA dimension across three tissue classes. Each row corresponds to one UDIP-FA dimension, and columns correspond to cerebrospinal fluid (CSF), grey matter (GM), and white matter (WM). Color intensity indicates the normalized Kolmogorov-Smirnov (K-S) statistic, with higher values indicating stronger enrichment of the corresponding UDIP-FA map in that tissue class. UDIP-FA, unsupervised deep imaging phenotypes of fractional anisotropy.

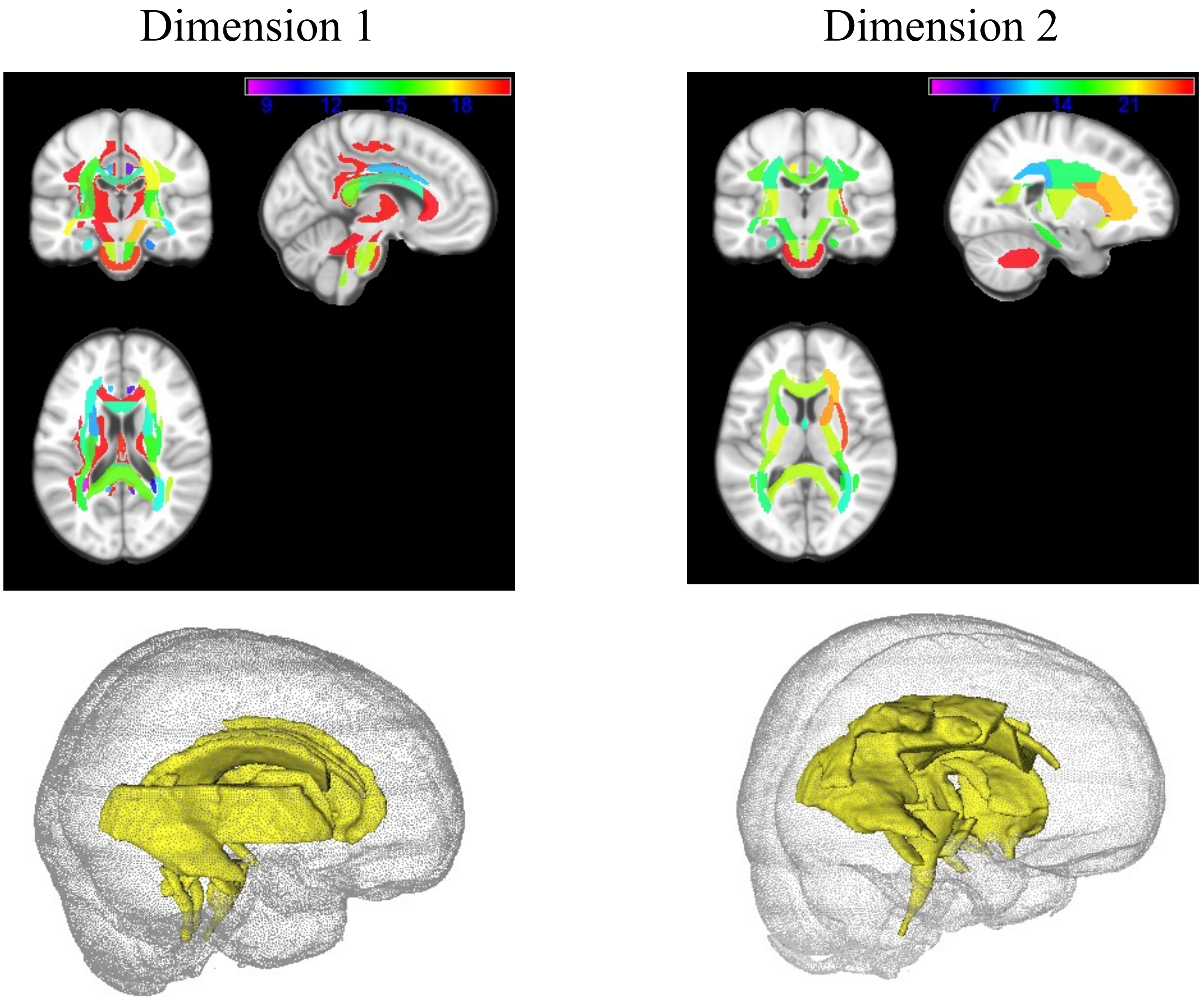

Supplementary Fig. 6 | Spatial enrichment patterns of UDIP-FA dimensions 1 and 2. Spatial visualization of two UDIP-FA dimensions using perturbation-based decoder interpretation (PerDI). The top row shows observed voxel-wise t values for UDIP-FA dimensions 1 and 2 across white matter (WM) tract regions. The bottom row shows WM regions significantly enriched for each dimension based on the PerDI-derived t maps and permutation testing with multiple-comparison correction. UDIP-FA, unsupervised deep imaging phenotypes of fractional anisotropy.

a

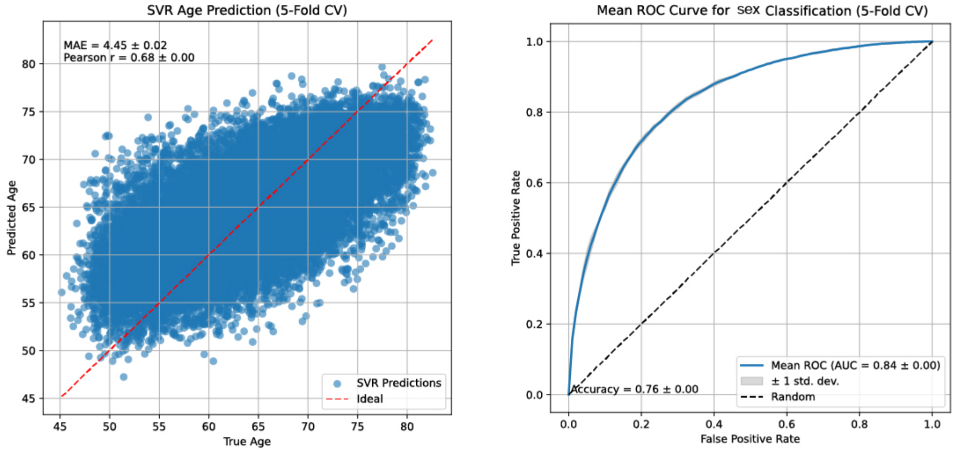

b

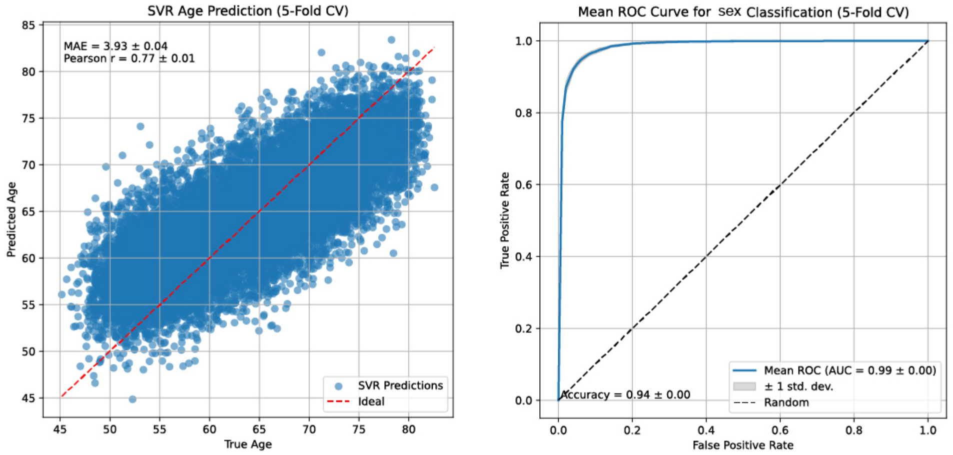

Supplementary Fig. 7 | Prediction of age and sex using traditional IDPs and UDIP-FA features. a, Prediction performance using traditional white matter (WM) tract imaging-derived phenotypes (IDPs). The left panel shows support vector regression (SVR) age prediction under five-fold cross-validation (CV), with predicted age plotted against chronological age; the red dashed line indicates perfect prediction. The right panel shows the mean receiver operating characteristic (ROC) curve for support vector machine (SVM) sex classification. Values are mean +/- s.d. across CV folds.

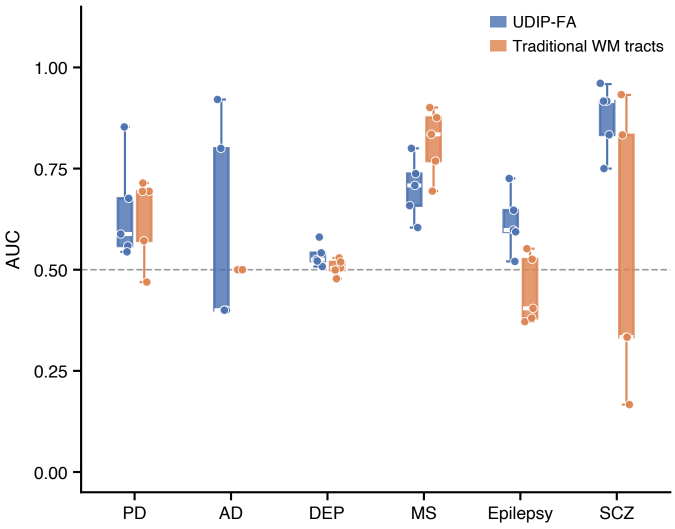

Supplementary Fig. 8 | Comparative disorder-classification performance of FA-derived phenotypes. Boxplots show the distribution of area under the receiver operating characteristic curve (AUC) values across five cross-validation folds for classification of six brain disorders using models trained on all fractional anisotropy (FA)-derived phenotypes (blue) or tract-averaged WM FA measures (orange). Center lines indicate medians, boxes indicate the interquartile range (IQR), and whiskers extend to the most extreme values within 1.5 x IQR. Points denote AUC values from individual folds, and the grey dashed line indicates chance-level performance (AUC = 0.5). PD, Parkinson's disease; AD, Alzheimer's disease; DEP, depression; MS, multiple sclerosis; EPI, epilepsy; SCZ, schizophrenia; WM, white matter.

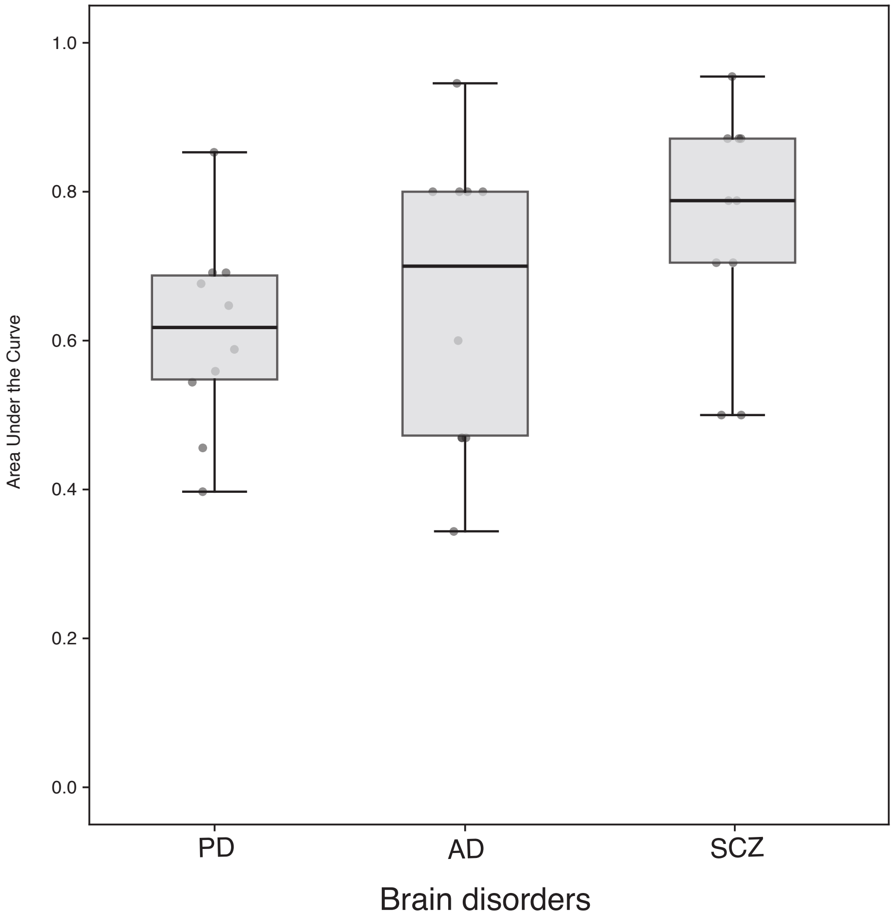

Supplementary Fig. 9 | Classification performance for smaller-sample disorders. Area under the receiver operating characteristic curve (AUC) values are shown for classification of Parkinson's disease (PD), Alzheimer's disease (AD), and schizophrenia (SCZ) using UDIP-FA features under five-fold cross-validation. Boxplots summarize AUC distributions across folds; center lines indicate medians, boxes indicate the interquartile range (IQR), and whiskers extend to 1.5 x IQR. Overlaid points denote individual cross-validation folds. UDIP-FA, unsupervised deep imaging phenotypes of fractional anisotropy.

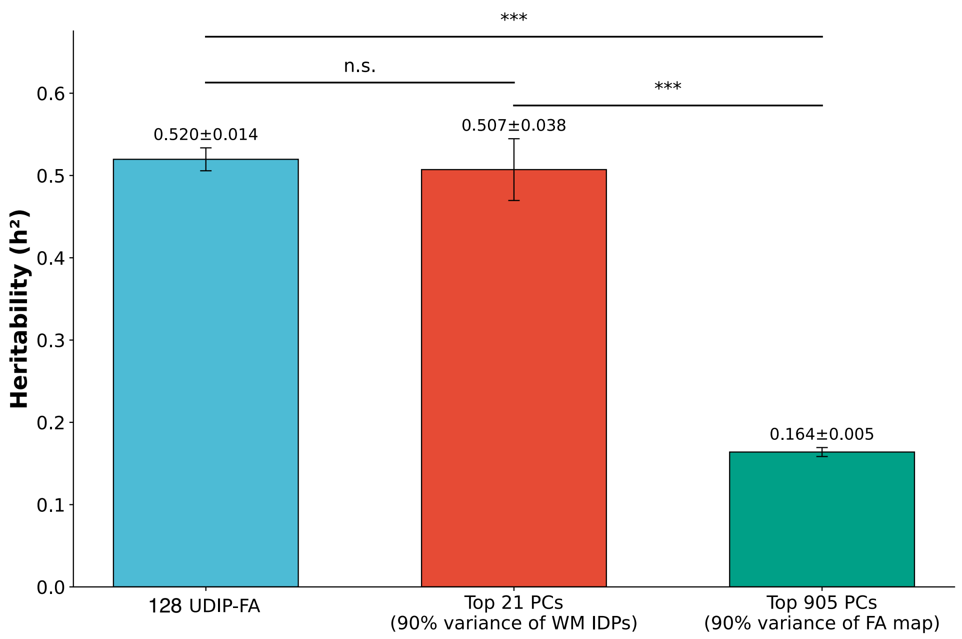

Supplementary Fig. 10 | Comparison of SNP heritability across FA feature representations. Bar plot showing single nucleotide polymorphism (SNP)-based heritability (h2) for three fractional anisotropy (FA) feature representations: 128 UDIP-FA latent features, the top 21 principal components (PCs) explaining 90% of the variance in white matter imaging-derived phenotypes (IDPs), and the top 905 PCs explaining 90% of the variance in whole-brain voxel-wise FA maps. Bars show point estimates with standard errors (s.e.), and numerical labels denote estimate +/- s.e. Horizontal brackets summarize pairwise comparisons using two-sided Mann-Whitney U tests. ***P < 0.001; n.s., not significant.

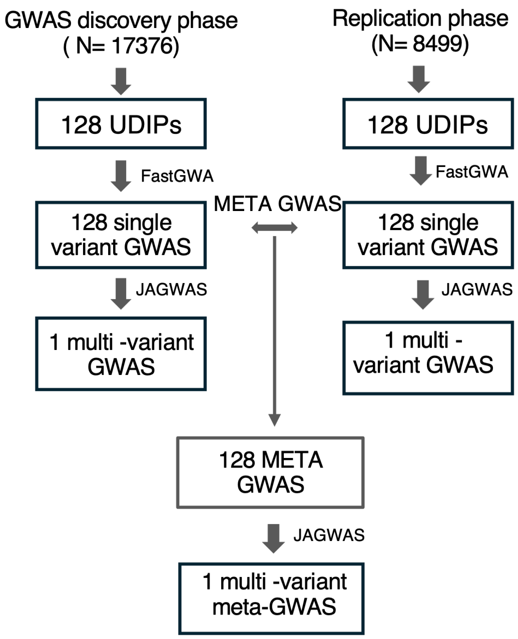

Supplementary Fig. 11 | Multi-stage GWAS workflow for UDIP-FA. Workflow for genome-wide association study (GWAS) analysis of UDIP-FA features. The study was divided into discovery (N = 17,376) and replication (N = 8,499) phases, each including 128 UDIP-FA dimensions. Single-variant GWAS was performed for each dimension in each phase, followed by multivariate GWAS using joint analysis of multiple phenotypes GWAS (JAGWAS). Meta-analysis was then conducted across discovery and replication single-variant results to generate 128 meta-GWAS summary statistics. Finally, multivariate meta-GWAS was performed using JAGWAS to identify joint genetic associations across UDIP-FA dimensions. UDIP-FA, unsupervised deep imaging phenotypes of fractional anisotropy.

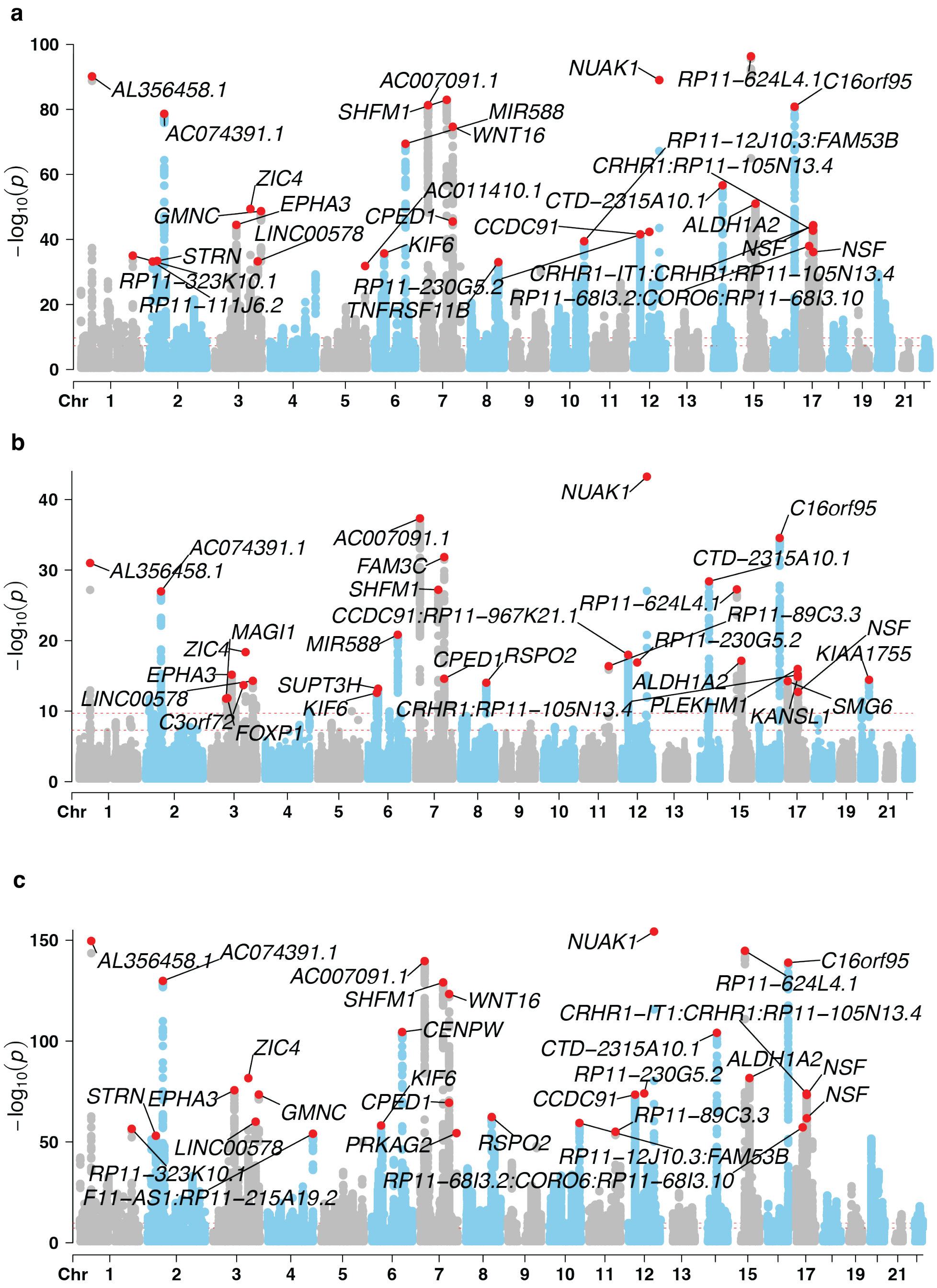

Supplementary Fig. 12 | Manhattan plots for multivariate GWAS of UDIP-FA. Manhattan plots show multivariate genome-wide association study (mvGWAS) results for UDIP-FA. a, Discovery cohort mvGWAS. b, Replication cohort mvGWAS. c, Meta-analysis combining the discovery and replication cohorts. Each point represents a single nucleotide polymorphism (SNP) plotted by genomic position and -log10(P) value. The top 30 most significant loci are annotated. UDIP-FA, unsupervised deep imaging phenotypes of fractional anisotropy.

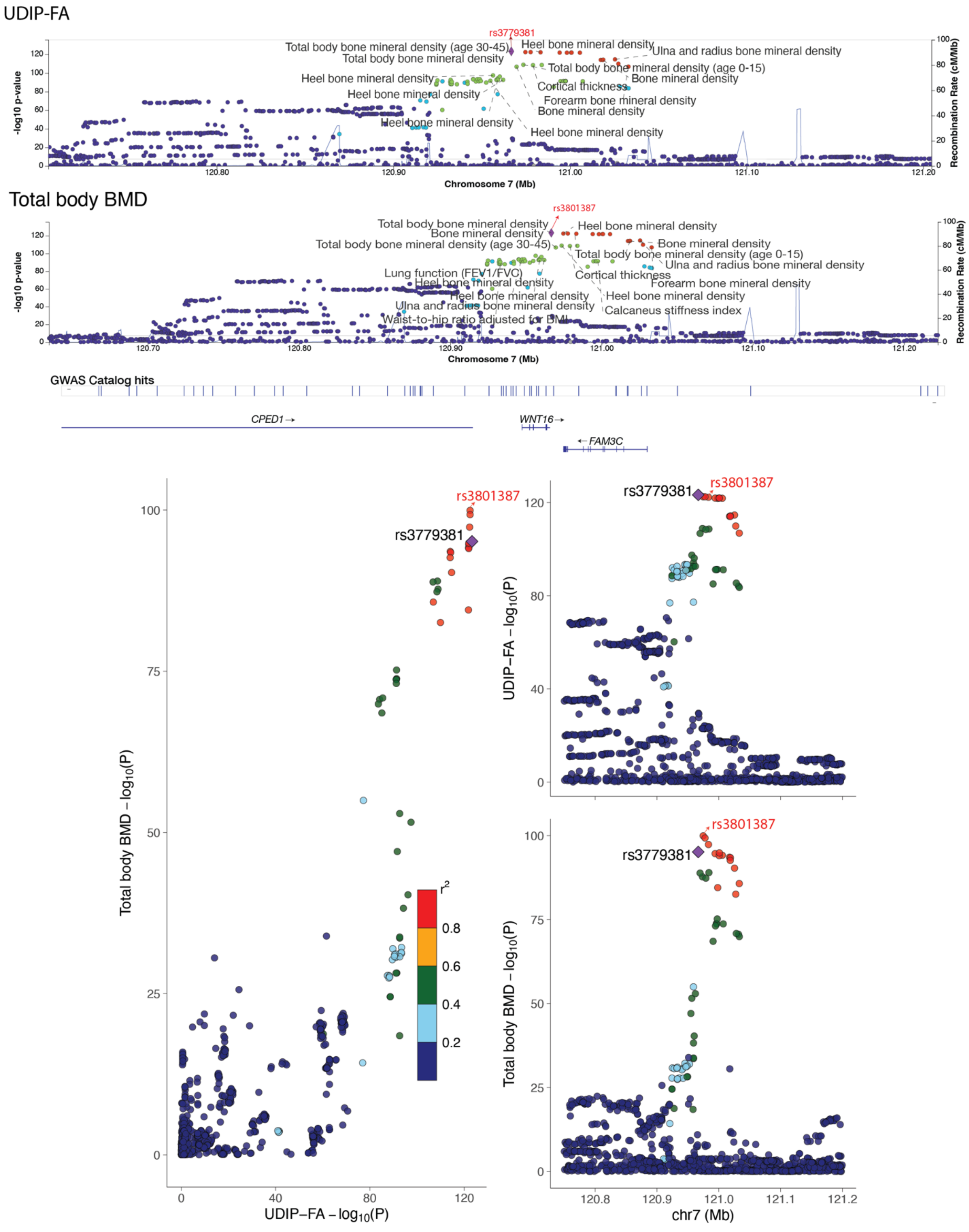

Supplementary Fig. 13 | Colocalized association signals for UDIP-FA and total body BMD at 7q31.31. Top, Regional association plots for UDIP-FA and total body bone mineral density (BMD) at the 7q31.31 locus, showing -log10(P) values by hg19 genomic position; lead single nucleotide polymorphisms (SNPs) are highlighted in red and recombination rate is shown in blue. Bottom left, Scatter plot comparing SNP -log10(P) values for UDIP-FA and total body BMD, colored by linkage disequilibrium (LD; r2) with rs3801387. Bottom right, zoomed plots from 120.8 to 121.2 Mb show a shared association peak near WNT16, with CPED1 and FAM3C annotated.

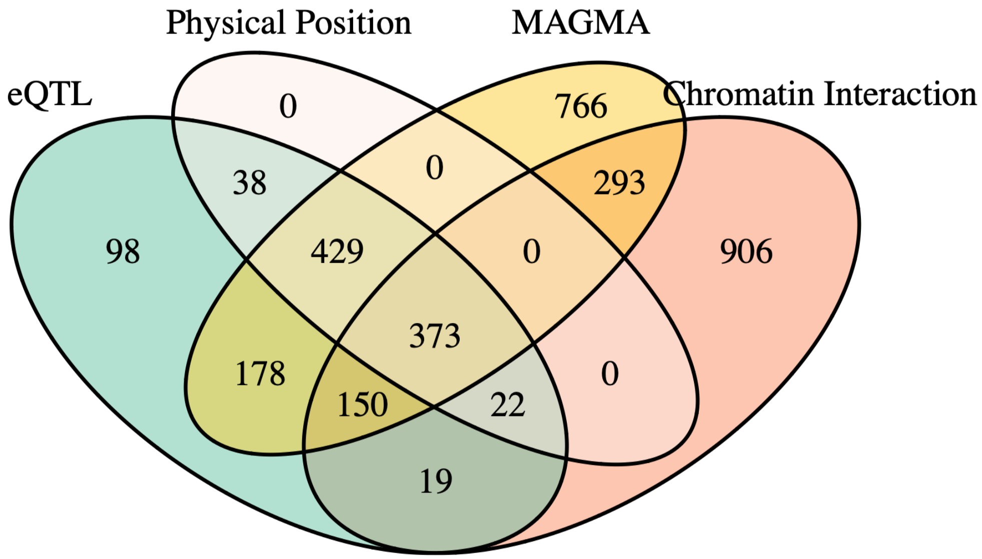

Supplementary Fig. 14 | Overlap of UFAGs identified by gene-mapping strategies. Venn diagram showing overlap among gene sets identified from the meta multivariate GWAS (meta-mvGWAS) of UDIP-FA using different gene-mapping strategies. Numbers indicate genes unique to, or shared across, the corresponding methods. eQTL, expression quantitative trait locus mapping; MAGMA, Multi-marker Analysis of Genomic Annotation.

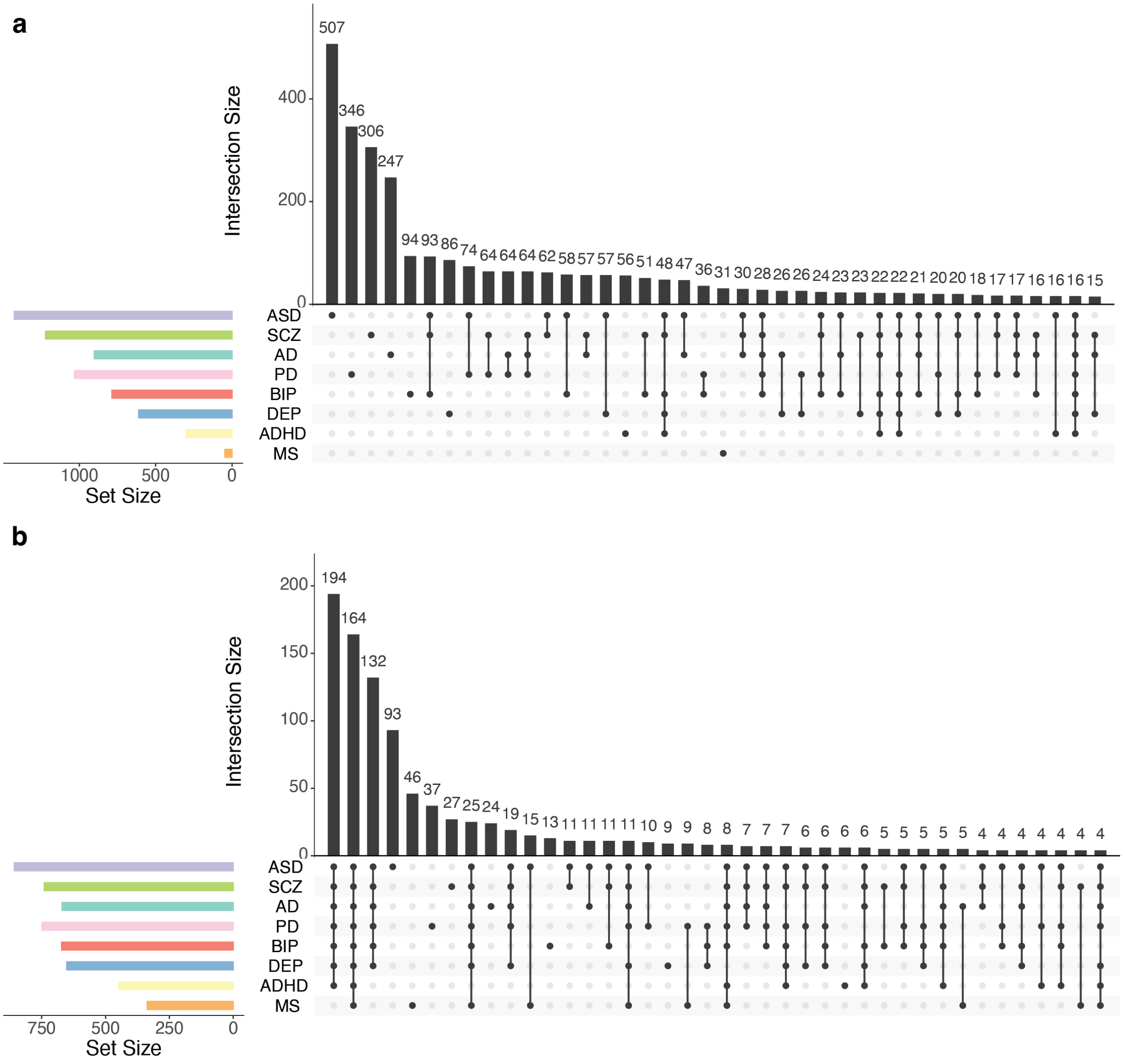

Supplementary Fig. 15 | Overlap of UFAG-interacting risk genes across brain disorders. UpSet plots show overlap among disease risk genes interacting with UDIP-FA-associated genes (UFAGs) in brain-specific molecular networks. a, Brain-specific transcriptional regulatory network (TRN). b, Brain-specific co-expression network. Bar charts above each matrix indicate intersection sizes, and horizontal bars on the left indicate set sizes for individual disorders. AD, Alzheimer's disease; ADHD, attention-deficit/hyperactivity disorder; ASD, autism spectrum disorder; BIP, bipolar disorder; DEP, depression; MS, multiple sclerosis; PD, Parkinson's disease; SCZ, schizophrenia.

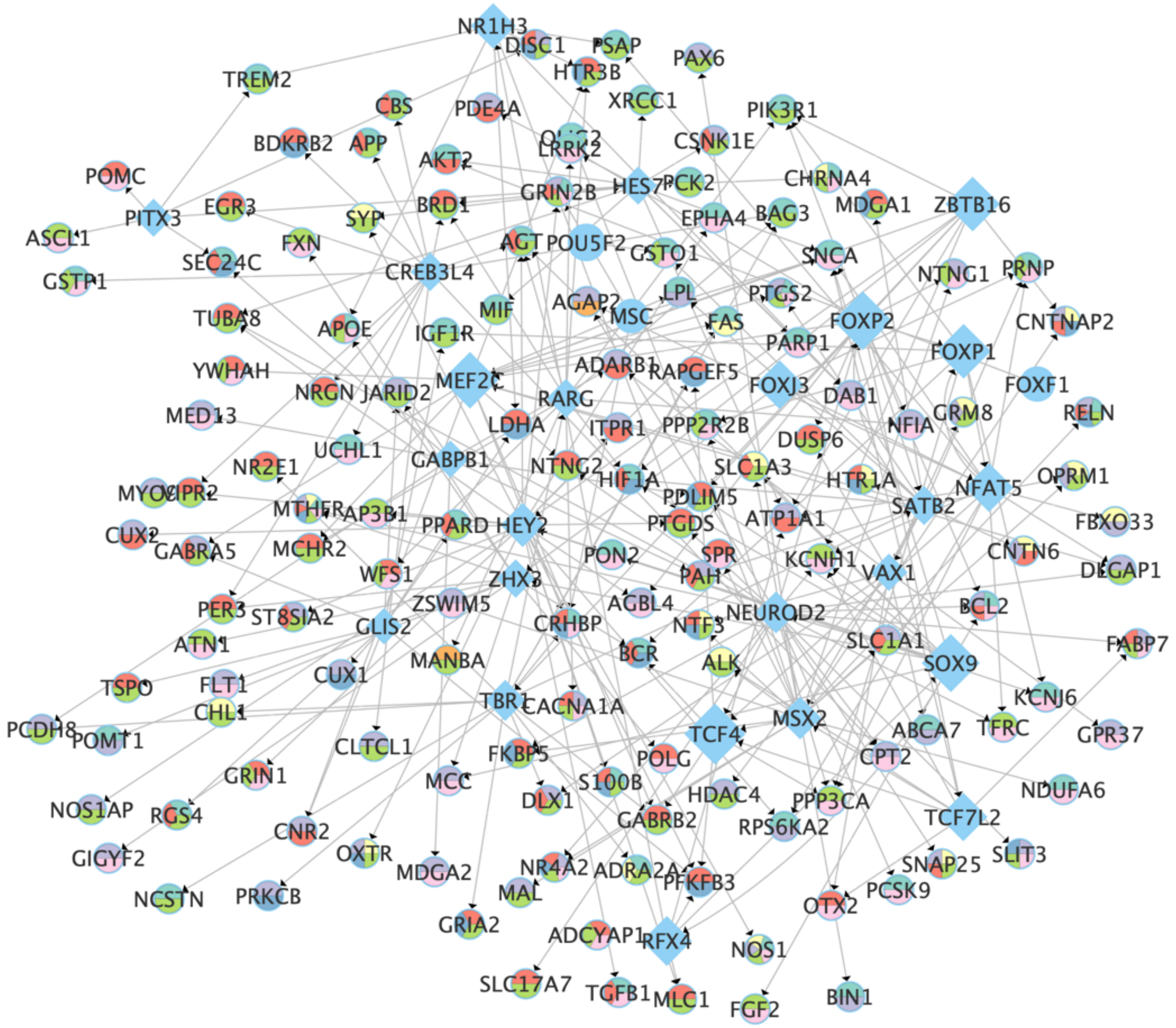

Supplementary Fig. 16 | Brain-specific TRN hub module linking UFAGs and disease risk genes. Network visualization of a pivotal hub module for UDIP-FA-associated genes (UFAGs) in the brain-specific transcriptional regulatory network (TRN). Diamonds represent UFAGs, circles represent brain disorder risk genes, and edges represent regulatory relationships. Pie-chart node colors indicate overlap across disorder-associated gene sets. Colors denote Alzheimer's disease (AD; mint green), attention-deficit/hyperactivity disorder (ADHD; pale yellow), autism spectrum disorder (ASD; lavender), bipolar disorder (BIP; coral red), depression (DEP; blue), multiple sclerosis (MS; orange), Parkinson's disease (PD; pink), and schizophrenia (SCZ; light green).

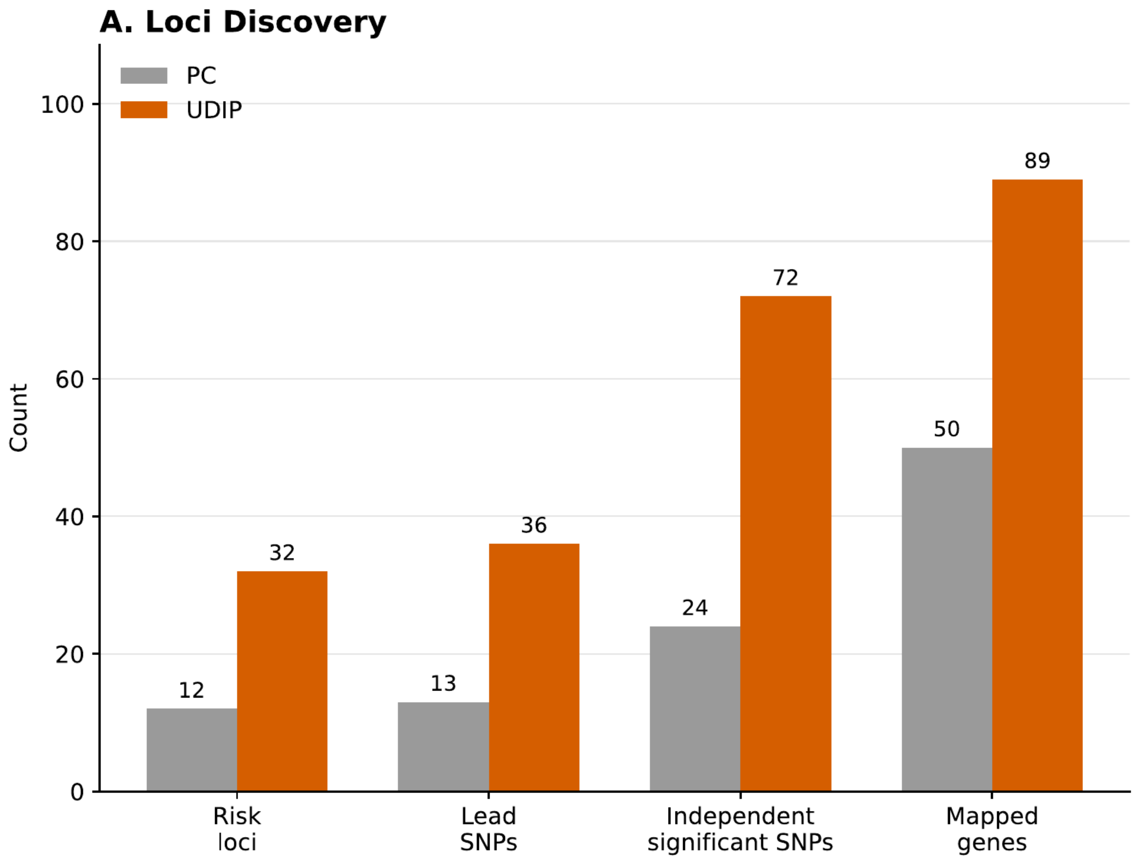

Supplementary Fig. 17 | Head-to-head comparison of loci discovery between PC-FA and UDIP-FA. a, Bar plot comparing genetic discovery between principal component (PC)-based traditional fractional anisotropy (FA) phenotypes and unsupervised deep imaging phenotypes of FA (UDIP-FA). Grey bars denote PC-based FA phenotypes, and orange bars denote UDIP-FA. The categories include risk loci, lead single nucleotide polymorphisms (SNPs), independent significant SNPs, and mapped genes. Numbers above bars indicate counts for each representation.

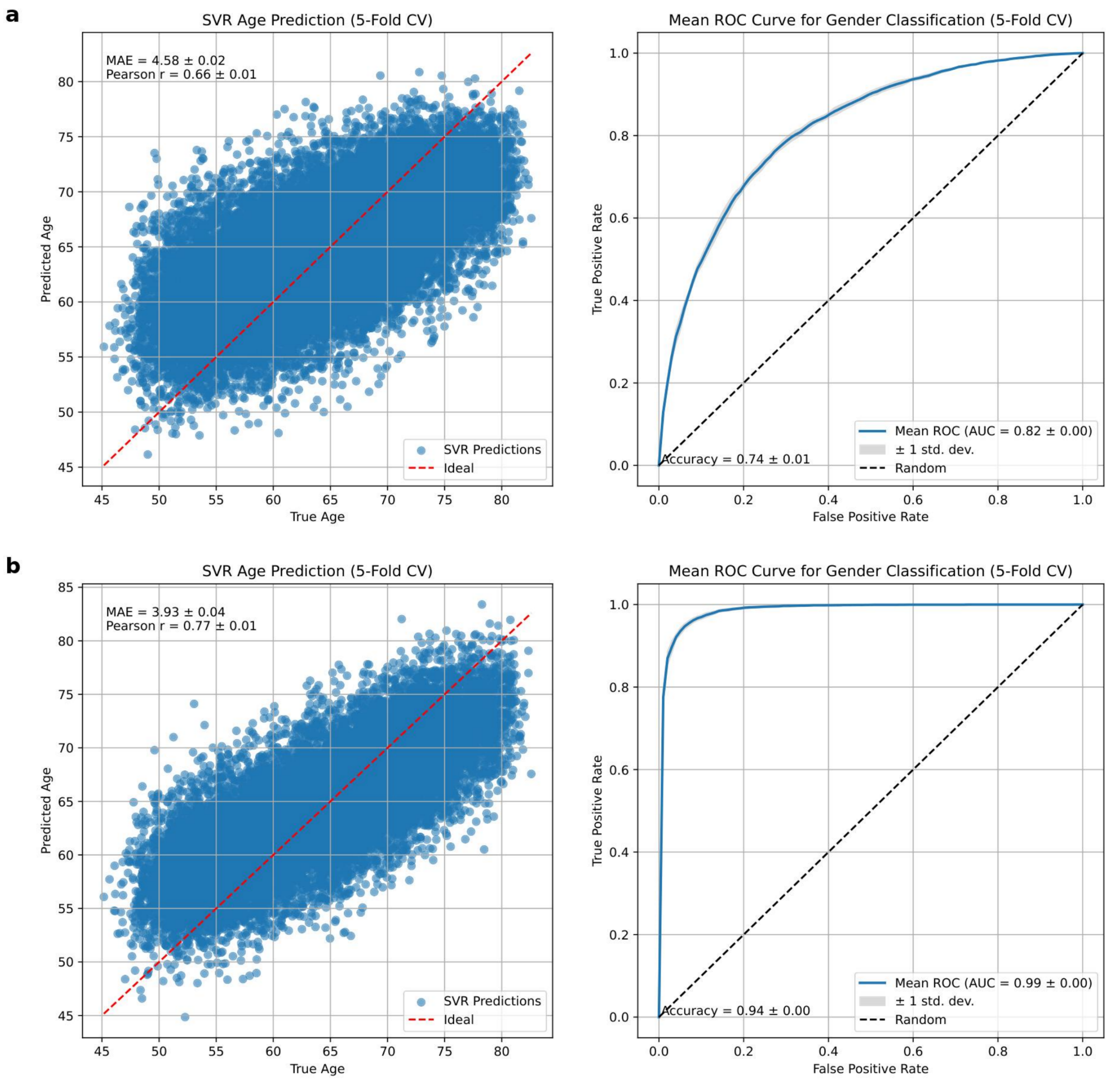

Supplementary Fig. 18 | Head-to-head comparison of trait prediction using PC and UDIP-FA features. a, Age prediction and sex classification using principal component (PC) features derived from traditional fractional anisotropy (FA) phenotypes. The left panel shows support vector regression (SVR) age prediction under five-fold cross-validation (CV), and the right panel shows the mean receiver operating characteristic (ROC) curve for sex classification. b, Corresponding prediction results using unsupervised deep imaging phenotypes of FA (UDIP-FA). The red dashed line indicates perfect age prediction, and the diagonal dashed line in ROC plots indicates random classification. MAE, mean absolute error; AUC, area under the ROC curve; s.d., standard deviation.

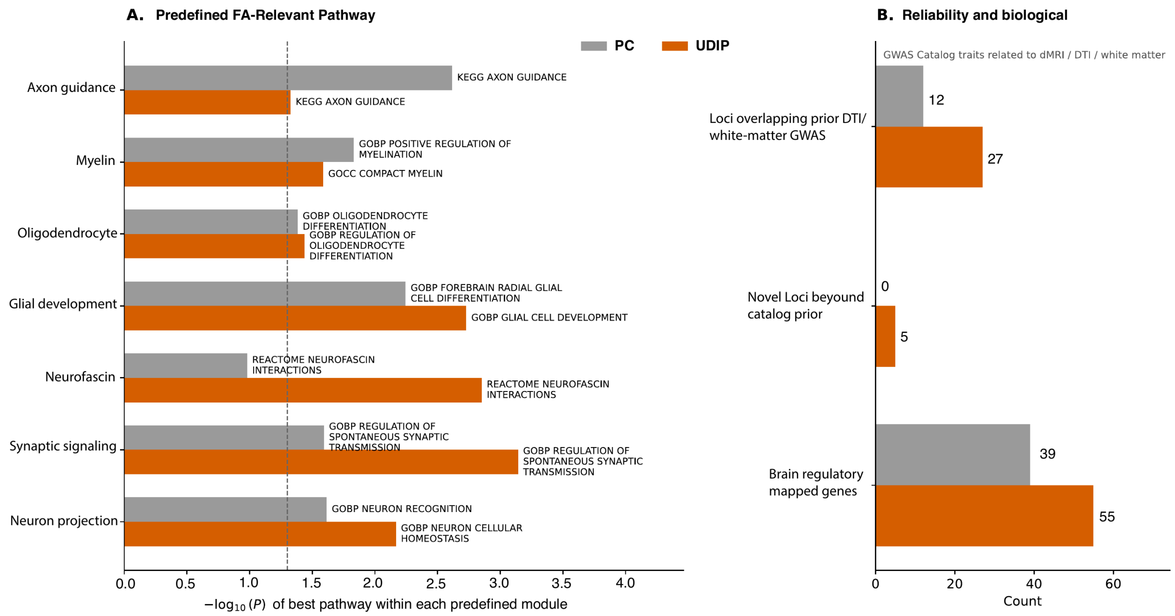

Supplementary Fig. 19 | Head-to-head comparison of PC and UDIP-FA in biological enrichment and reliability. a, Comparison of predefined FA-relevant pathway enrichment between principal component (PC)-based FA phenotypes and unsupervised deep imaging phenotypes of FA (UDIP-FA). Bars show -log10(P) for the best pathway within each predefined biological module, including axon guidance, myelin, oligodendrocyte, glial development, neurofascin, synaptic signaling, and neuron projection modules. b, Reliability and biological relevance comparison based on counts of loci overlapping prior diffusion magnetic resonance imaging (dMRI), diffusion tensor imaging (DTI), or white matter genome-wide association studies (GWAS), loci beyond prior GWAS Catalog evidence, and brain regulatory mapped genes. Grey bars denote PC-based features, and orange bars denote UDIP-FA.

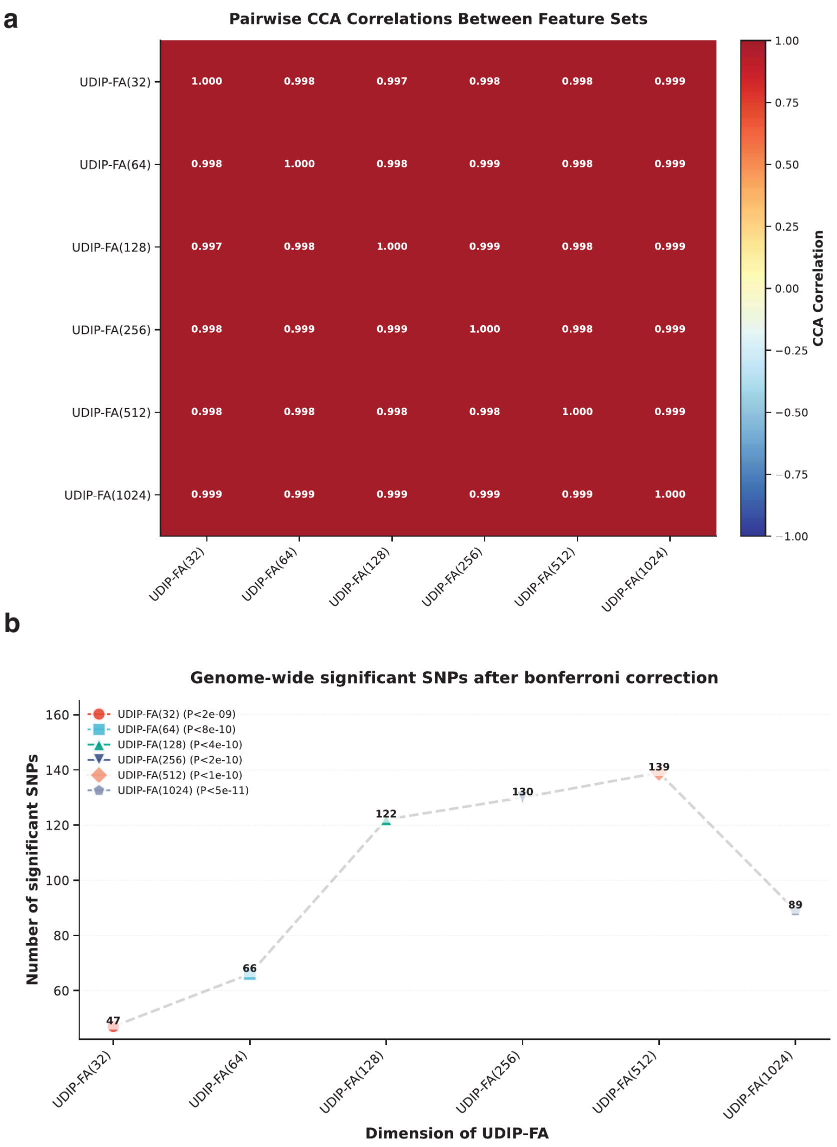

Supplementary Fig. 20 | Stability and model selection of UDIP-FA across latent dimensions. a, Pairwise canonical correlation analysis (CCA) heatmap showing similarity among UDIP-FA feature sets extracted from models with different latent dimensions (32, 64, 128, 256, 512, and 1024). High CCA correlations indicate stable latent structure across model sizes. b, Sensitivity analysis of genome-wide significant single nucleotide polymorphism (SNP) discovery across latent dimensions after Bonferroni correction. The 128-dimensional UDIP-FA model selected for the main analysis provides robust discovery power while maintaining model parsimony. UDIP-FA, unsupervised deep imaging phenotypes of fractional anisotropy.
