# Supplementary material for: Genetic architecture of white matter microstructure captured by unsupervised deep representation learning of fractional anisotropy maps
Source: Nat Commun. 2026 Jun 3;17:7150. doi: 10.1038/s41467-026-73996-z (PMC13396798; doi:10.1038/s41467-026-73996-z)
Supplement: Supplementary file 2 — Description of Additional Supplementary Files [file 41467_2026_73996_MOESM2_ESM.pdf]

Supplementary Data

File name: Supplementary Data 1

Description: Regression results quantifying the variance in conventional tract-level FA measurements explained by the 128 UDIP-FA features across 48 UK Biobank white matter tracts.

File name: Supplementary Data 2

Description: SNP-based heritability estimates for the 128 UDIP-FA features, including statistical significance and multiple-testing correction results.

File name: Supplementary Data 3

Description: Discovery-replication genetic correlation results for single-variant GWAS of the 128 UDIP-FA features estimated using LD score regression.

File name: Supplementary Data 4

Description: Lead SNPs and associated genomic loci identified in the discovery-cohort multivariate GWAS of UDIP-FA.

File name: Supplementary Data 5

Description: Replication results for discovery-cohort lead SNPs, including replication-cohort association statistics and Bonferroni-corrected significance information.

File name: Supplementary Data 6

Description: Lead SNPs and genomic loci identified in the final meta-analysis multivariate GWAS of UDIP-FA.

File name: Supplementary Data 7

Description: NHGRI-EBI GWAS Catalog lookup results for UDIP-FA-associated lead SNPs, including previously reported trait associations.

File name: Supplementary Data 8

Description: MAGMA gene-based association results for the meta-analysis multivariate GWAS summary statistics.

File name: Supplementary Data 9

Description: Functional mapping results linking significant variants to candidate genes using positional mapping, eQTL mapping, and chromatin-interaction mapping.

File name: Supplementary Data 10

Description: Phenotype and disease enrichment results for UDIP-FA-associated genes generated using ToppGene.

File name: Supplementary Data 11

Description: MAGMA gene-set enrichment results based on MSigDB gene sets for UDIP-FA-associated genetic signals.

File name: Supplementary Data 12

Description: External GWAS summary statistics and trait information used to construct polygenic risk scores for association testing with UDIP-FA.

File name: Supplementary Data 13

Description: Curated risk gene sets for brain disorders used in overlap and network analyses.

File name: Supplementary Data 14

Description: Brain-active protein-protein interaction network data and UFAG-disease risk gene network interaction results used for molecular network analyses.

File name: Supplementary Data 15

Description: Drug-gene interaction results from DGIdb for UDIP-FA-associated genes, restricted to approved drugs and mechanism-defined interactions relevant to downstream therapeutic analyses.
