# Supplementary material for: Genetic architecture of white matter microstructure captured by unsupervised deep representation learning of fractional anisotropy maps
Source: Nat Commun. 2026 Jun 3;17:7150. doi: 10.1038/s41467-026-73996-z (PMC13396798; doi:10.1038/s41467-026-73996-z)
Supplement: Supplementary file 4 — Reporting Summary [file 41467_2026_73996_MOESM4_ESM.pdf]

nature portfolio

Corresponding author(s): Degui Zhi

Last updated by author(s): Apr 27, 2026

Reporting Summary

Nature Portfolio wishes to improve the reproducibility of the work that we publish. This form provides structure for consistency and transparency in reporting. For further information on Nature Portfolio policies, see our Editorial Policies and the Editorial Policy Checklist.

Statistics

For all statistical analyses, confirm that the following items are present in the figure legend, table legend, main text, or Methods section.

n/a	Confirmed	
☐	☒	The exact sample size (*n*) for each experimental group/condition, given as a discrete number and unit of measurement
☐	☒	A statement on whether measurements were taken from distinct samples or whether the same sample was measured repeatedly
☐	☒	The statistical test(s) used AND whether they are one- or two-sided<br>*Only common tests should be described solely by name; describe more complex techniques in the Methods section.*
☐	☒	A description of all covariates tested
☐	☒	A description of any assumptions or corrections, such as tests of normality and adjustment for multiple comparisons
☐	☒	A full description of the statistical parameters including central tendency (e.g. means) or other basic estimates (e.g. regression coefficient) AND variation (e.g. standard deviation) or associated estimates of uncertainty (e.g. confidence intervals)
☐	☒	For null hypothesis testing, the test statistic (e.g. *F*, *t*, *r*) with confidence intervals, effect sizes, degrees of freedom and *P* value noted<br>*Give P values as exact values whenever suitable.*
☒	☐	For Bayesian analysis, information on the choice of priors and Markov chain Monte Carlo settings
☒	☐	For hierarchical and complex designs, identification of the appropriate level for tests and full reporting of outcomes
☐	☒	Estimates of effect sizes (e.g. Cohen's *d*, Pearson's *r*), indicating how they were calculated

Our web collection on statistics for biologists contains articles on many of the points above.

Software and code

Policy information about availability of computer code

Data collection	Data collections of all using datasets were not performed by us.
Data analysis	For the data analysis, we used Python v3.9, R v4.1.0, pytorch v1.10, Cytoscape v3.8.0, LDSC (LD SCore) v1.0.1, MAGMA v1.08, FUMA, the python package of nilearn v0.8.1, numpy v1.21.2, pandas 1.3.4, and the R packages of clusterProfiler v3.16.1, Matrix v1.2-18, gdata v2.18.0, tidyverse v1.3.1, ggpubr v0.4.0, and VennDiagram v1.6.20. Custom code that supports the findings of this study is available here (https://github.com/ZhiGroup/UDIP-FA.). Additional information related to this paper may be requested from the authors.

For manuscripts utilizing custom algorithms or software that are central to the research but not yet described in published literature, software must be made available to editors and reviewers. We strongly encourage code deposition in a community repository (e.g. GitHub). See the Nature Portfolio guidelines for submitting code & software for further information.

Data

Policy information about availability of data

All manuscripts must include a data availability statement. This statement should provide the following information, where applicable:

- Accession codes, unique identifiers, or web links for publicly available datasets
- A description of any restrictions on data availability
- For clinical datasets or third party data, please ensure that the statement adheres to our policy

Our using dataset were all from UK biobank, which was accessed via approved project 24247.

Research involving human participants, their data, or biological material

Policy information about studies with human participants or human data. See also policy information about sex, gender (identity/presentation), and sexual orientation and race, ethnicity and racism.

Reporting on sex and gender	The term sex (biological attribute, i.e., female or male) was used in this study.
Reporting on race, ethnicity, or other socially relevant groupings	Not applicable for this study.
Population characteristics	Not applicable for this study.
Recruitment	Data collections of all using datasets were not performed by us.
Ethics oversight	Not applicable for this study.

Note that full information on the approval of the study protocol must also be provided in the manuscript.

Field-specific reporting

Please select the one below that is the best fit for your research. If you are not sure, read the appropriate sections before making your selection.

☒ Life sciences ☐ Behavioural & social sciences ☐ Ecological, evolutionary & environmental sciences

For a reference copy of the document with all sections, see nature.com/documents/nr-reporting-summary-flat.pdf

Life sciences study design

All studies must disclose on these points even when the disclosure is negative.

Sample size	For training this model, a dataset of 6130 images from subjects of mixed ethnicities was chosen as the model development set. And for the GWAS analysis, we adopt 25875 samples to do the GWAS analysis on the extracted UDIP
Data exclusions	For UKBB participants have strong kinship, and without MR image data and were excluded, details show in Methods.
Replication	No replication was conducted given the huge sample size of the present study
Randomization	Not applicable for this study.
Blinding	Investigators were not blind to the datasets.

Reporting for specific materials, systems and methods

We require information from authors about some types of materials, experimental systems and methods used in many studies. Here, indicate whether each material, system or method listed is relevant to your study. If you are not sure if a list item applies to your research, read the appropriate section before selecting a response.

Materials & experimental systems

n/a	Involved in the study	
☒	☐	Antibodies
☒	☐	Eukaryotic cell lines
☒	☐	Palaeontology and archaeology
☒	☐	Animals and other organisms
☒	☐	Clinical data
☒	☐	Dual use research of concern
☒	☐	Plants

Methods

n/a	Involved in the study	
☒	☐	ChIP-seq
☒	☐	Flow cytometry
☐	☒	MRI-based neuroimaging

Magnetic resonance imaging

Experimental design

Design type	Not applicable for this study.
Design specifications	Not applicable for this study.

Behavioral performance measures	Not applicable for this study.

Acquisition

Imaging type(s)	Diffusion-weighted images from UKBB
Field strength	3 Tesla
Sequence & imaging parameters	Diffusion-weighted images were acquired using a single-shot spin-echo EPI sequence with multiband acceleration (×3) and a monopolar Stejskal–Tanner diffusion preparation (2×2×2 mm; b=1000/2000 s/mm², 50 directions each; additional b0 and reverse-phase b0 volumes).
Area of acquisition	whole brain

Diffusion MRI ☒ Used ☐ Not used

Parameters: Diffusion-weighted images were acquired using a single-shot spin-echo EPI sequence with multiband acceleration (×3) and a monopolar Stejskal–Tanner diffusion preparation (2×2×2 mm; b=1000/2000 s/mm², 50 directions each; additional b0 and reverse-phase b0 volumes).

Preprocessing

Preprocessing software	The diffusion MRI data in UKBB were primarily acquired using Siemens Skyra 3T MRI scanners operating VD13A SP4, equipped with a standard Siemens 32-channel RF head coil. The diffusion-weighted images (DWIs) utilized a multi-shell diffusion sequence, optimized for consistent quality across multiple imaging centers. To maximize generalizability and reduce feature engineering, we followed the straightforward preprocessing pipeline provided by the UKBB imaging team, primarily utilizing the FMRIB Software Library (FSL; https://www.fmrib.ox.ac.uk/ukbiobank/). Key preprocessing steps provided by UKBB include correction for motion and eddy current distortions using FSL's eddy tool, diffusion tensor fitting using FSL's DTIFIT, and generation of bias-field-corrected FA images. Subsequently, all FA images were spatially normalized to the MNI152 space provided by UKBB via linear registration using FSL FNIRT. Normalization ensured the standardization of head sizes and alignment of brain structures across subjects, while preserving relevant structural deformation information
Normalization	linear registration to MNI space.
Normalization template	MNI space
Noise and artifact removal	Not applicable for this study.
Volume censoring	Not applicable for this study.

Statistical modeling & inference

Model type and settings	Not applicable for this study.
Effect(s) tested	Not applicable for this study.

Specify type of analysis: ☒ Whole brain ☐ ROI-based ☐ Both

Statistic type for inference (See Eklund et al. 2016)	Not applicable for this study.
Correction	Not applicable for this study.

Models & analysis

n/a	Involved in the study
☒	☐ Functional and/or effective connectivity
☒	☐ Graph analysis
☐	☒ Multivariate modeling or predictive analysis

Multivariate modeling and predictive analysis	We adopted a deep learning model for extracting the representation of FA maps by the preprocessing diffusion MR images. Details show in Methods
