# Supplementary material for: Genetic architecture of white matter microstructure captured by unsupervised deep representation learning of fractional anisotropy maps
Source: Nat Commun. 2026 Jun 3;17:7150. doi: 10.1038/s41467-026-73996-z (PMC13396798; doi:10.1038/s41467-026-73996-z)
Supplement: Supplementary file 5 — Transparent Peer Review file [file 41467_2026_73996_MOESM5_ESM.pdf]

nature portfolio

Peer Review File

Genetic architecture of white matter microstructure captured by unsupervised deep representation learning of fractional anisotropy maps

Corresponding Author: Professor Degui Zhi

This file contains all reviewer reports in order by version, followed by all author rebuttals in order by version.

Version 0:

Reviewer comments:

Reviewer #1

(Remarks to the Author)
In this paper, Zhao et al. applied a deep neural network based dimension reduction method to extract lower dimensional representation of whole brain voxelwise fractional anisotropy, and then examine their associations with genetics and clinical phenotypes in UKBiobank. The authors have done an extensive work on how those 128 derived lower representation values, i.e. UDIP-FA, are associated with age, sex, clinical phenotypes, single genetic variants, and polygenic scores, aiming to showcase that UDIP-FA is a better metric than anatomically defined ROIs. While this is a remarkable feat, the fundamental question is whether UDIP-FA is indeed a better alternative than ROI based approach. Similar to other multivariate methods, UDIP suffers from the interpretability issue. The authors have to recourse back to use "enrichment" analysis to see which ROIs are most likely to be the source of the association found by UDIP associations, defeating the argument that UDIP-FA is a better tool than anatomically defined ROIs. Although UDIP has the potential to improve the power to detect loci, as many other multivariate methods have done, it suffers from the biases and confounds more, as the core algorithm does not ward against any influence of confounds and biases in the data. The weakness of such deep learning unsupervised approach cannot be ignored.

There are also other concerns I hope the authors can address:
Major:
1. In the prediction for age, sex, and disorders, the models are fit with different algorithms without clear justification, leaving the impression of cherry picking results. Meanwhile, the AUC for the disorders is not a good metric in current context. UKBiobank imaging set is known to be biased toward overtly healthy, therefore, the labels are heavily skewed toward controls. Skewed labeling will lead to over optimistic AUC, despite poor accuracy in predicting cases. One telling sign of this issue is that Depression has the highest rate in the population, but their predictive performance is the worst. Schizophrenia, on the other hand, has the lowest prevalence rate in the population, but their predictive performance is the highest.

2. The key analysis of this paper relies on a multivariate GWAS method (JAGWAS) that has not been peer reviewed. In its preprint, there is no simulations or proof to indicate that JAGWAS can effectively control the type I error, despite known issue for applying MANOVA in the high-dimension setting. It is questionable how valid the abundant loci discovered are valid.

Minor:
1. What exactly is the acronym UDIP stands for? It showed up very early in the abstract and introduction, without explicitly spelling it out first.

2. It is unclear what UMAP can provide given that their 128 UDIP dimensions are relatively independent to each other.

3. The association figures have a lot of overlapping dots and texts, making it very hard to parse (Figure 3 and 5).

(Remarks on code availability)
The code base looks valid with detailed instruction.

Reviewer #2

(Remarks to the Author)
This work builds on the work from the group (Patel et al. Commun Biol 2024) and introduces an auto-encoder (deep learning) framework, UDR-WM, that extracts high-dimensional brain-wide FA features, termed UDIP-FAs, from voxel-wise FA maps. The resulting features, called UDIP-FAs, are used to explore the genetic architecture of WM microstructure and its links to complex brain traits. A multivariate Genome-Wide Association Study (GWAS) identified 939 significant lead SNPs in 586 loci. The associated genes (UFAGs) are overexpressed in glial cells, particularly astrocytes and oligodendrocytes, and are genetically correlated with multiple brain disorders. This is an exciting unsupervised learning (deep learning) representation of white matter and presents some interesting results. I congratulate the authors on their efforts.

I do have a few concerns that are not clear in the current version.

Major:

1) Biological Interpretation:
While the paper attempts to interpret the high-dimensional UDIP-FA features using the PerDI approach and regional enrichment, the complex, distributed patterns captured by the 128 dimensions remain a challenge for full, intuitive biological interpretation compared to simpler, atlas-defined measures.
I found the R2 values are reported for the 48 major tracts, and the UDIP-FA enrichment patterns across different WM tracts for dimension 1 and dimension 2 (UMAP embedding). In addition, some select UDIP-FA dimensions are described in lines 145-154. However, I do not seem to find enrichments for the 128 UDIP-FA features in the main text or supplements. Connecting the major WM tracts to these 128 UDIP-FA features could add some biological interpretation to each of these features, for example, UDIP-FA feature x could show enrichment like UMAP dimension 1 or 2, or could be widespread. In addition, such enrichment or something on those lines could be a stepping stone for the adoption of these high-dimensional UDIP-FA features by the readers working with the major WM tracts.

2) FA features:
For me, it seems that the high-dimensional UDIP-FA features are a mix of white matter integrity, brain morphology, and anthropometric measures. While overlap is expected, it's the ranking of overlap that seems to indicate that UDIP-FA's may be capturing other features, above white matter microstructure.

a) In the section "Comparison of UDIPs across different brain imaging modalities320", Lines 332-333: "Furthermore, loci identified across different models revealed a consistent pattern: T1 and FA shared 374 loci (80.09% overlap), while T2 shared 399 loci (86.18%) with the same set." This suggests UDIP-FA features capture a strong brain morphometric component.

b) In the section "Comparison with previous GWAS studies", in table S7, "Overlap of UDIP-FA–associated SNPs with the GWAS Catalog", shows anthropometric measures (e.g., Height, Waist circumference adjusted for body mass index, etc.) and brain morphology (e.g., Cortical surface area, Brain morphology (MOSTest), Cortical thickness, etc.) with most occurrences. The white matter trait that appears most is "White matter microstructure (radial diusivities)", with 118 occurrences. While indirect and dependent on the GWAS Catalog count, this seems to point to UDIP-FA may be capturing beyond FA features, including other DTI-derived measures.
It will help the readers if this could be quantified. For example, using the approach mentioned in lines 106-108: "Specifically, we extracted average FA values from 48 major white matter tracts defined by the UK Biobank and used multiple linear regression to assess the extent to which the 128 UDIP-FA features explained variance in each tract (Methods)." Here, the comparative measures could be global measures including FA, MD, RD, total SA, cortical thickness, ICV, BMI, and height, and one can assess the extent to which the 128 UDIP-FA features explain variance in such metrics.

In a scenario where these UDIP-FA go beyond FA, appropriate changes in the terminology and other parts of the paper may be warranted.

3) Predictive power:
In Lines 132-134, the authors mention: "UDIP-FA demonstrated strong sensitivity to age (mean absolute error (MAE) = 3.93 133 ± 0.04, Pearson's r = 0.77 ± 0.01; Figure 2c), outperforming a previously published study that used the traditional FA value from different WM regions[15]."
Since the training and test sample sizes could play a role in model evaluation, it would be appropriate if a baseline age prediction model using traditional FA value from different WM regions is used on the same training and test set.

4) Heritability comparison:
In Lines 163-168, authors mention: "A total of 115 UDIP-FAs (89.8%) were significantly heritable (FDR-corrected P <0.05), with heritability estimates ranging from 6.9% to 85.2% (mean = 52.0%; Figure 3A and Table S2, (30.5%) UDIP-FAs showed high heritability ($h2$ > 0.6). The heritability of our UDIP-FAs was significantly higher than that of a previous study (mean $h2$ =

34.9%, P < 2.2e-16, Mann–Whitney U test, Figure 3b). This is remarkable as our UDIP-FAs are largely uncorrelated."

While the statistics seem correct.
I have a simple question: since these 128 UDIP-FA can be seen as a dimensionality reduction of the input FA maps and are largely uncorrelated, would an apple-to-apple comparison of heritability estimates involve comparing the heritability estimates derived from (for example) the top N principal components (>90% variance) across the WM tracts or input measures?

5) Interpreting the Gene-based analysis:

While remarkable, the sheer number of "3,480" UFAGs seems too high to draw any specific conclusions.
For example, in lines 309-310: "This analysis revealed that 44 UFAGs interact with 87 drugs used to treat 55 distinct brain-related diseases (Table S15)". While specific examples are highlighted, could the authors guide the potential readers on addressing potential false positives?

While multivariate analysis of multiple measures may enhance the discovery of genetic variants, including MOSTest, van der Meer Nature Comm 2020, follow-up on such a large set creates another set of challenges. As such, providing a guide to the reader with not only specific examples, but also navigating false positives, would help the adoption of UDIP-FA.

Minor:

1) Lines 103-105: "As shown in Figure 2a, UDIP-FA representations captured meaningful population-level variation, with FA values exhibiting a smooth gradient across the latent space."This gradient is not as standout as Fig 2 in ref [13], Patel et al, 2024, and the gradient only seems to be along the x-axis. If it helps, could you please explore the possibility of marginal density/histogram plots to illustrate the gradient point?

2) Lines 135-138: "In addition, we evaluated the ability of UDIP-FA to predict the status of six brain disorders: Alzheimer’s Disease (AD), Depression (DEP), Epilepsy (EPI), Multiple Sclerosis (MS), Parkinson’s Disease (PD), and Schizophrenia (SCZ). Patients with these conditions were identified from the UK Biobank (UKB) dataset using ICD-10 diagnostic codes (field 41270, Methods)." The sample size (case-control) information is only provided for MS cases on line 521. Please add the same for other disorders.

3) Terminology for disorders. If I may suggest, please use neurological and psychiatric disorders for neurological (AD, PD, etc.) and psychiatric disorders (behaviourally defined, SCZ, DEP, etc.). For example, line 203: "for several neurological disorders, including schizophrenia (SCZ)33"

4) In the section "UDIP-FA demonstrates predictive power for age, sex, and neurological disorders", the baseline for comparison could be a standard model with major WM tract measures. Since the case and control groups are imbalanced, and training involves sampling an equal number of cases, it would be more appropriate to provide a baseline model for comparison.

5) In the section "UDIP-FA demonstrates predictive power for age, sex, and neurological disorders", the most prominent UDIP-FA features for AD, MS, and EPI are described. However, the exclusion of SCZ, the one with the highest AUC, seems perplexing. If the UDIP-FA features are more widespread for SCZ, that would be interesting. This difference could reflect the neurological and psychiatric disorders.

6) The number of risk genes, Table S13, specifically for ASD, a staggering 968 genes, seems perplexing. See, for example, Wagstyl et al. eLife (2024) https://doi.org/10.7554/eLife.86933.3. Would the findings remain similar if curated disease gene sets from other published work were explored?

(Remarks on code availability)
The code appears to be comprehensive and easy to follow, covering most aspects of the analysis, but a detailed assessment may be needed.

Reviewer #3

(Remarks to the Author)
Overall, this is a solid and well-executed study. The methodology is clearly described, the analyses are comprehensive, and the results are biologically plausible and internally consistent. The manuscript convincingly demonstrates the potential of unsupervised deep representations for imaging-genetics research.

That said, I have a few questions and points that would benefit from further clarification or additional validation:

1) Stability and reproducibility of embeddings: Have the authors evaluated how stable the UDIP-FA latent space is across different random initializations, training runs, or subsets of data? Are the same latent dimensions consistently recovered, or is there variability between models?

2) External validation: Can the pretrained model be applied to other cohorts or scanners? If so, how well do the embeddings generalize, and what procedures (if any) are needed to align new data to the UDIP-FA space?

(Remarks on code availability)
While the study provides extensive downstream analysis code, the repository currently lacks the full training and inference pipeline for the autoencoder model used to derive the UDIP-FA embeddings.

For reproducibility and broader utility, it would be important for the authors to release or document in detail the complete procedure—from preprocessing of raw FA maps to generation of UDIP-FA features—including model architecture, training parameters, and inference steps.

In addition, a public script or clear instructions to project new cohorts into the pretrained UDIP-FA space would enable independent validation and cross-cohort applications, which are essential for evaluating the robustness and generalizability of the proposed framework.

Version 1:

Reviewer comments:

Reviewer #1

(Remarks to the Author)
In this revision, Zhao et al. had addressed most of my concerns. Some minor suggestions:

1. While Figure R1 is informative in a way by comparing to more traditional PC approach, it is a bit off-target. The key metrics they used to justify UDIP is a). loci discovery, b). trait predictions, and c). genetic signal enrichment. However, none of those are compared head-to-head comparison.

2. Though I disagree on the interpretation on how AUC varies according to prevalence, I can accept the way authors revised the manuscript and tried matching schema to mitigate the unbalanced design.

3. It is good for authors to compare their JAGWAS to other methods such as MOSTest. However, the correlations between p-values let the skewed extreme cases dominate the evaluation while ignore those divergent cases in the P = E^-10~20 range (Figure R1). It still does not answer a critical question - does JAGWAS control type I error sufficiently given the significance threshold they imposed?

(Remarks on code availability)

Reviewer #2

(Remarks to the Author)
The authors have made substantial revisions addressing many reviewer concerns. I appreciate their efforts. However, some concerns remain that require attention before publication.

1) UDIP-FA demonstrates predictive power for neurological and psychiatric disorders:
The authors added the sample size of each disorder and compared their model with a baseline model using standard measures from major white matter (WM) tracts. The authors report SCZ achieves the highest AUC despite having only 36 cases. This is paradoxical and suggests potential overfitting. While the authors argue in their rebuttal (REF1.2 – Prediction of UDIP-FA response) that this reflects "biological signal strength" rather than a statistical artifact, this interpretation is not adequately supported. With 128 features and only 36 cases, even with downsampling and cross-validation, the risk of spurious feature importance rankings is substantial.

Suggestion: Either (a) provide support to show that for small sample sizes the results reflect biological signal and not statistical artifact, (b) restrict analyses to disorders with adequate sample sizes (DEP, EPI, MS), or (c) clearly state in the main text that results for SCZ, AD, and PD are exploratory and underpowered, moving detailed interpretation to supplementary materials.

2) On a related point, in the same section, the feature Importance Interpretation from small samples needs some validity.

lines 162-165: "In AD, the critical UDIP-FA feature (dimension_7) was significantly enriched in the left cingulum (cingulate gyrus) (CGC-L), the genu of the corpus callosum (GCC), and the splenium of the corpus callosum (SCC), with a Bonferroni-corrected $P < 0.05$ based on permutation test results from PerDI."

These interpretations are derived from models trained on 63 AD cases. Feature importance rankings from gradient boosting with 128 input features and ~60 cases are highly unstable. The authors cite literature showing these tracts are "implicated in

AD-related pathology," but this may reflect the model picking up on well-established group differences that would be detectable with conventional methods.

Suggestion: Add stability analyses (e.g., bootstrap confidence intervals on feature importance rankings) or acknowledge explicitly that these interpretations are hypothesis-generating only and require validation in larger samples.

3) External Validation and clinical utility.
(Also raised in REF3.3 – External validation of UDIP-FA)

Lines 454-459 acknowledge this limitation:
"while our discovery-replication design confirmed internal reproducibility under standardized UK Biobank acquisition, we have not yet assessed the model's generalization to external cohorts with varying scanners or protocols"

For a method proposed as capturing "biologically and clinically informative brain phenotype" (Line 30-31), the absence of external validation is a limitation.

Suggestion: Please rephrase claims about clinical utility throughout. Specifically, revise the Abstract (Lines 30-33) and Discussion to position this as a proof-of-concept requiring external validation before clinical translation claims are warranted.

Minor:
1) Shared variance between UDIP-FA and global structural and anthropometric phenotypes.
The authors added Figure S4, showing the proportion of variance in the 128 UDIP-FA features explained by different sets of imaging-derived and anthropometric measures, estimated using canonical correlation analysis (CCA).
And added text on Lines 120-124, Lines 441-445, and Lines 715-717.

This explanation is plausible, but raises the question on interpretation of "the biological specificity of UDIP-FA" (Lines 120-121). If UDIP-FA captures global brain development signals, is it truly providing WM-specific insights, or is it a general brain structure phenotype?

Suggestion: Add a sentence in the Discussion explicitly addressing whether the shared variance with cortical morphology is a limitation for WM-specific applications or an advantage for capturing integrated brain structure.

(Remarks on code availability)
The code appears to be comprehensive and easy to follow, covering most aspects of the analysis, but a detailed assessment may be needed.

Open Access This Peer Review File is licensed under a Creative Commons Attribution 4.0 International License, which permits use, sharing, adaptation, distribution and reproduction in any medium or format, as long as you give appropriate credit to the original author(s) and the source, provide a link to the Creative Commons license, and indicate if changes were

made.
In cases where reviewers are anonymous, credit should be given to 'Anonymous Referee' and the source.
The images or other third party material in this Peer Review File are included in the article’s Creative Commons license, unless indicated otherwise in a credit line to the material. If material is not included in the article’s Creative Commons license and your intended use is not permitted by statutory regulation or exceeds the permitted use, you will need to obtain permission directly from the copyright holder.
To view a copy of this license, visit https://creativecommons.org/licenses/by/4.0/

Response Letter to

Unveiling genetic architecture of white matter microstructure through unsupervised deep representation learning of fractional anisotropy maps

REF1 – Point-to-Point response to REF1

REF1.1 – Interpretability of UDIP-FA

Referee Comment	*Major Comments:*<br>*In this paper, Zhao et al. applied a deep neural network based dimension reduction method to extract lower dimensional representation of whole brain voxelwise fractional anisotropy, and then examine their associations with genetics and clinical phenotypes in UKBiobank. The authors have done an extensive work on how those 128 derived lower representation values, i.e. UDIP-FA, are associated with age, sex, clinical phenotypes, single genetic variants, and polygenic scores, aiming to showcase that UDIP-FA is a better metric than anatomically defined ROIs. While this is a remarkable feat, the fundamental question is whether UDIP-FA is indeed a better alternative than ROI based approach. Similar to other multivariate methods, UDIP suffers from the interpretability issue. The authors have to recourse back to use "enrichment" analysis to see which ROIs are most likely to be the source of the association found by UDIP associations, defeating the argument that UDIP-FA is a better tool than anatomically defined ROIs. Although UDIP has the potential to improve the power to detect loci, as many other multivariate methods have done, it suffers from the biases and confounds more, as the core algorithm does not ward against any influence of confounds and biases in the data. The weakness of such deep learning unsupervised approach cannot be ignored.*
Author Response	Thanks for your comment. We agree that it is crucial to clarify (i) how UDIP-FA compares to conventional ROI-based FA IDPs, (ii) how its interpretability is achieved, and (iii) how we guard against confounding and bias.<br><br>(1) **Clarifying the role of UDIP-FA relative to ROI-based IDPs**<br><br>Our intention is not to position UDIP-FA as a replacement for anatomically defined ROIs, but rather as a complementary representation that captures fine-grained, voxel-level microstructural variation that is not fully summarized by ROI averages. We have revised the Introduction and Discussion to remove language suggesting that UDIP-FA is “better” in a generic sense and instead emphasize that UDIP-FA provides additional information beyond traditional ROI FA IDPs while remaining compatible with standard ROI-based analyses.

To address the question about comparative performance, we systematically compare UDIP-FA to a set of ROI-based FA metrics under matched conditions. Specifically:

a) Using a held-out test set, we first show that the 128 UDIP-FA dimensions explain substantially more variance in whole-brain voxelwise FA values than the 49 ROI-averaged FA phenotypes (IDPs) derived from the FA images (Figure R1a). We further examined the relationship between ROI-averaged FA measures and individual UDIP-FA dimensions and found that ROI-based measures exhibit much stronger inter-regional correlations than UDIP-FA, indicating higher redundancy (Figure R1b). Moreover, UDIP-FA requires a larger number of principal components to capture the same level of variance, reflecting greater feature diversity. Together, these results demonstrate that UDIP-FA retains significantly more independent and fine-grained information from the original FA maps, even when compared at similar dimensionality (Figure R1c).

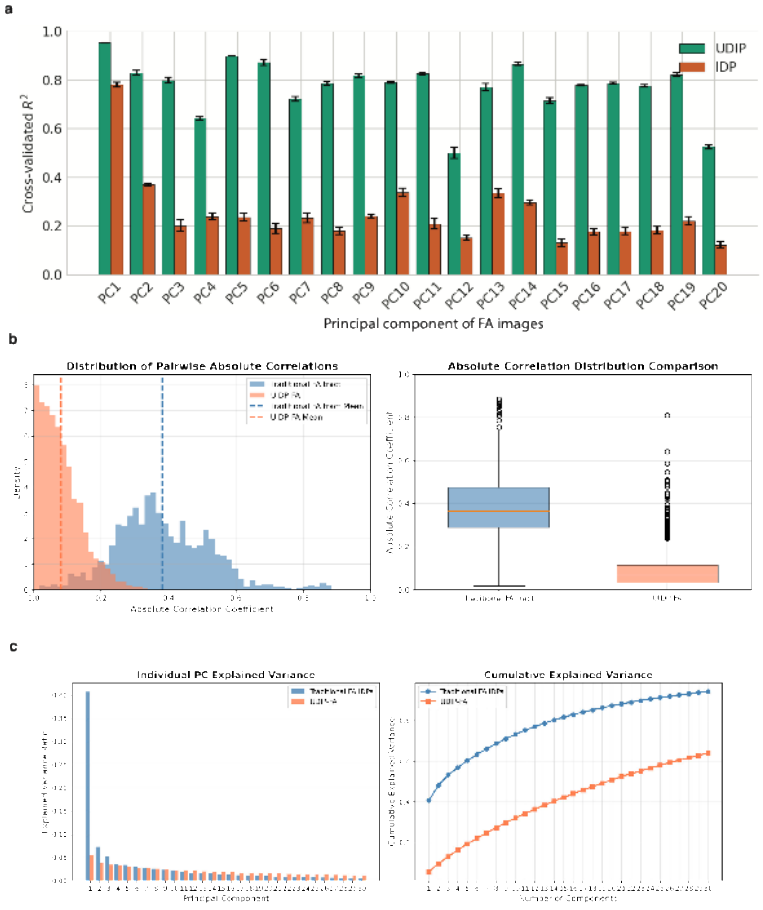

Figure R1 | UDIP-FA captures substantially richer and more diverse information than traditional FA tract IDPs. (a) Cross-validated

	prediction performance (R^2) for the first 20 principal components (PCs) of FA images using UDIP-FA features versus traditional FA tract IDPs. UDIP-FA consistently achieves markedly higher R^2 across nearly all PCs, indicating that UDIP-FA retains a substantially larger portion of the underlying variance in voxelwise FA patterns. **(b)** Comparison of feature redundancy between UDIP-FA and traditional FA tract IDPs. Left: Distribution of pairwise absolute correlations shows that IDPs exhibit strong collinearity, whereas UDIP-FA features have much lower correlations, reflecting higher feature diversity. Right: Boxplot comparison highlights the significantly reduced correlation structure within UDIP-FA, demonstrating that UDIP-FA provides a more decorrelated and information-rich representation. **(c)** Information content comparison via principal component analysis of UDIP-FA features and WM IDPs. Left: Individual explained variance ratios show that early PCs from UDIP-FA account for more variance than those derived from IDPs. Right: Cumulative explained variance curves confirm that UDIP-FA captures the variance structure of FA images more efficiently and with fewer components.<br><br>b) We find that the UDIP-FA show that for age, sex, UDIP-FA achieve higher cross-validated prediction performance than models based on traditional ROI WM tracts (test on same samples). This result suggests that the UDIP-FA can better characteristic the biological function (Figure S7).

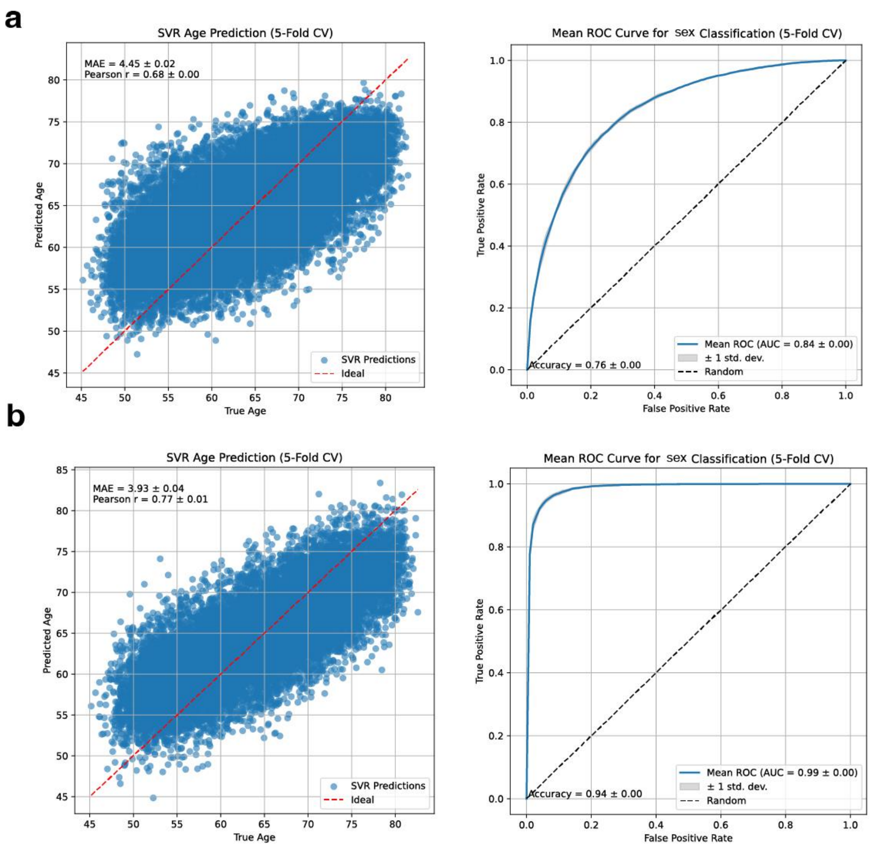

Figure S7 | Comparison of prediction performance between traditional WM tracts (IDPs) and UDIP-FA features. (a) Prediction performance using traditional white-matter tract IDPs. Left: Support Vector Regression (SVR) results for age prediction under 5-fold cross-validation, showing the relationship between predicted and true age (MAE = 4.45 ± 0.02; Pearson's r = 0.68 ± 0.00). Right: Support Vector Machine (SVM) Mean ROC curve for sex classification across 5 folds (AUC = 0.84 ± 0.00; accuracy = 0.76 ± 0.00) (b) Prediction performance using UDIP-FA features. Left: SVR-based age prediction under 5-fold cross-validation demonstrates improved accuracy relative to traditional IDPs (MAE = 3.93 ± 0.04; Pearson's r = 0.77 ± 0.01). Right: Sex classification ROC curve showing substantially enhanced discriminative ability (AUC = 0.99 ± 0.00; accuracy = 0.94 ± 0.00).

These results support the claim that UDIP-FA provides increased statistical power and richer brain-wide information relative to conventional ROI-based FA IDPs, while we now frame this improvement as "added value beyond ROI measures" rather than an absolute replacement.

(2) Interpretability of UDIP-FA

	To further validate UDIP-FA beyond extensive biological analyses, we incorporated a deep-learning interpretability framework to assess its ability to characterize white-matter fiber tracts. Specifically, we used PerDI to decompose the image regions activated by each UDIP dimension and compared these activation profiles with traditional tract-based measures. Traditional fiber tracts, which are well-established and biologically interpretable, served as orthogonal evidence to evaluate the alignment between UDIP-FA features and known white-matter anatomy. This analysis demonstrates that individual UDIP-FA dimensions capture information distributed across multiple WM tracts, reinforcing UDIP-FA's capability to represent biologically meaningful white-matter structure. To facilitate broader exploration, we also developed an interactive web tool that visualizes UDIP-FA's tract-level representations (https://udip-fa.github.io/Dim_visualization/).<br><br>**(3) Methods to avoid confounding and bias**<br><br>We implemented several strategies to minimize potential confounding and bias throughout the imaging and genetic analyses. First, all input images underwent uniform preprocessing, ensuring consistent calibration and standardization across participants and imaging sites. In the GWAS analyses, we explicitly included relevant covariates, including site information to adjust for residual variation. The LDSC intercepts for all individual GWAS were close to 1, indicating minimal inflation and little evidence of confounding. Additionally, for multi-GWAS procedures and phenotypic correlation estimation, we regressed covariates from all phenotypes and constructed the covariance matrix from the resulting residuals. This approach substantially reduces the influence of shared confounders and further supports the robustness of our findings.<br><br>In the revision, we have revised the Abstract, Introduction and Discussion to clarify that UDIP-FA is intended as a complementary, data-driven representation that refines and extends conventional ROI-based FA phenotypes rather than replacing them. In addition, we have expanded the Discussion of limitations to more explicitly acknowledge the interpretability trade-offs and the potential sensitivity of high-capacity unsupervised models to residual confounds, despite extensive covariate adjustment and LDSC-based quality control. We also outline future directions, such as confound-robust representation learning and validation in independent cohorts, to further address these issues.
Excerpt 1.1 (main text)	Line 30-33<br><br>"Together, our findings establish UDIP-FA as a biologically and clinically informative brain phenotype that complements conventional ROI-based FA

	measures, enabling a higher-resolution dissection of WM genetic architecture and its genetic links to complex traits"<br><br>Line 70-81<br><br>"Unlike traditional ROI-based approaches that aggregate signals within predefined anatomical boundaries, UDIP-FA leverages full voxel-wise information to capture spatially distributed and continuous microstructural patterns. Consequently, we position UDIP-FA not as a replacement for anatomically defined ROIs, but as a complementary, high-dimensional phenotype that captures distinct, fine-grained variance in white matter microstructure. We then performed a multistage, multivariate GWAS of UDIP-FAs to delineate their genetic architecture. The UDIP-FA enabled us to elucidate the functional and biological mechanisms associated with FA and to investigate the associations between UDIP-FA and brain disorders at both the phenotypic and molecular levels. Our findings suggest that UDIP-FA serves as a robust global imaging phenotype, offering enhanced sensitivity for capturing the genetic architecture of WM and its links to psychiatric and neurological disorders.<br>"<br><br>Line 447-453<br><br>"However, we acknowledge that while UDIP-FA provides a complementary, fine-grained representation, this data-driven approach entails a trade-off between voxel-level sensitivity and the direct anatomical interpretability of traditional ROIs. Although our PerDI approach offers a partial solution, further improvements are needed. Second, regarding potential data confounding, although we applied rigorous covariate adjustment to minimize bias, unsupervised deep learning representations can remain sensitive to residual scanner or physiologic effects"

REF1.2 – Prediction of UDIP-FA

Referee Comment	*Major Comments:*<br><br>*In the prediction for age, sex, and disorders, the models are fit with different algorithms without clear justification, leaving the impression of cherry picking results. Meanwhile, the AUC for the disorders is not a good metric in current context. UKBiobank imaging set is known to be biased toward overtly healthy, therefore, the labels are heavily skewed toward controls. Skewed labeling will lead to over optimistic AUC, despite poor accuracy in predicting cases. One telling sign of this issue is that Depression has the highest rate in the population, but their predictive performance is the worst.*

	Schizophrenia, on the other hand, has the lowest prevalence rate in the population, but their predictive performance is the highest.
Author Response	Thank you for your comments. We apologize for any confusion regarding our model selection and validation strategy. We wish to clarify that the primary objective of this analysis was not to develop a clinical-grade diagnostic model, but rather to demonstrate the biological informativeness and sensitivity of UDIP-FA in capturing disease-relevant signals. Below, we clarify our rationale for the chosen algorithms and specifically address the concerns regarding AUC interpretation and class imbalance.<br><br>**1. Justification for algorithm selection**. We did not intend to "cherry pick" algorithms to inflate results. Rather, we selected standard, task-appropriate algorithms based on the nature of the target variable:<br><br>a) Age: This is a continuous variable, so we employed Support Vector Regression (SVR), a standard approach for regression tasks.<br>b) Sex: This is a balanced binary classification task, for which we used a Support Vector Machine (SVM), widely recognized for its robustness in high-dimensional binary classification.<br>c) Disorders: This is a complex binary classification task with significant class imbalance. We adopted Gradient Boosted Decision Trees (GBDT) because they are highly effective at handling non-linear relationships and are robust to potential overfitting in complex datasets.<br><br>**2. Class imbalance and the validity of AUC**. We respectfully disagree with the premise that our AUC scores are inflated by skewed labeling. The reviewer is correct that the UK Biobank is biased toward healthy controls; however, we explicitly designed our training pipeline to neutralize this bias before the model learned from the data.<br><br>a) Training Strategy (Down-sampling): As detailed in the Methods section, we utilized a stochastic down-sampling strategy. Within each cross-validation fold, we randomly down-sampled the majority class (controls) to match the exact number of the minority class (cases). Consequently, the model was trained on a perfectly balanced (1:1) dataset. It was physically impossible for the model to achieve "optimistic" performance by simply predicting the majority class or learning prior probabilities based on population skew.<br>b) AUC Validity: Because our training data was balanced, the AUC remains a valid and robust metric. Unlike Accuracy (which can be misleadingly high in imbalanced datasets if the model simply predicts "Healthy"), AUC measures the model's ability to rank a randomly chosen positive instance higher than a randomly chosen

	negative instance, independent of the class distribution in the test set.<br><br>**3. Biological interpretation of Depression vs. Schizophrenia performance**. The reviewer notes that Depression (highest prevalence) had the worst performance, while Schizophrenia (lowest prevalence) had the highest. We believe this observation validates our model's ability to detect true pathological signals, rather than being an artifact of skew. If the model were biased by prevalence (skewed labeling), it would likely perform better (or exhibit higher bias) on the more common condition (Depression) and fail to detect the rare condition (Schizophrenia). The fact that the inverse occurred suggests the results are driven by biological signal strength:<br><br>a) Schizophrenia (high signal): Schizophrenia is a neurodevelopmental disorder associated with profound, widespread white matter dysconnectivity[1]. Our study identified strong enrichment of UDIP-FAs in genes like SOX10 and MOBP (essential for myelination). Despite the small sample size (low prevalence), the structural "signal" in white matter is strong, allowing the model to discriminate cases effectively.<br>b) Depression (low signal): Depression is biologically heterogeneous with more subtle white matter alterations. Despite the larger sample size (high prevalence), the structural signal is weaker and more variable, resulting in lower predictive performance.<br><br>In summary, the variation in AUC reflects the distinct underlying neuropathology of these disorders captured by UDIP-FA, rather than artifacts of population prevalence. We have revised the manuscript to make this justification and our handling of the data imbalance clearer.
Excerpt 1.2 (main text)	Line 568-580<br><br>"To evaluate the biological informativeness of UDIP-FA, we selected algorithms tailored to the specific nature of each target variable: Support Vector Regression (SVR) was used for age prediction (continuous), Support Vector Machines (SVM) for sex classification (balanced binary), and Gradient Boosted Decision Trees (GBDT) implemented by LightGBM for brain disorder classification, owing to their robustness in handling complex non-linear interactions. To address the significant class imbalance (healthy-volunteer bias) and ensure the validity of the AUC metric, we implemented a balanced training strategy for the disorder classification tasks. Within each cross-validation fold, we performed stochastic down-sampling of the majority class (controls) to match the exact number of cases, ensuring that the models were trained on unbiased data. Model performance was evaluated on each held-out test fold using the area under the receiver operating characteristic curve (AUC), and the mean AUC across the five

	folds was reported. Feature importance was determined using LightGBM's gain-based ranking, averaged across folds to identify robust predictive markers"<br><br>Line 366-369<br><br>"We utilized predictive modeling tasks, such as brain age estimation and disease association, primarily as a proof-of-concept to validate the biological informativeness of these features, rather than to establish a clinical diagnostic model, particularly given the sample size constraints for specific disorders"

REF1.3 – Validity of JAGWAS

Referee Comment	*Major Comments:*<br><br>*The key analysis of this paper relies on a multivariate GWAS method (JAGWAS) that has not been peer reviewed. In its preprint, there is no simulations or proof to indicate that JAGWAS can effectively control the type I error, despite known issue for applying MANOVA in the high-dimension setting. It is questionable how valid the abundant loci discovered are valid.*
Author Response	Thank you for your comments. We would like to respectfully clarify that JAGWAS is still a single-variant GWAS approach that performs a chi-square–based multi-trait association test for each genetic variant separately for millions of genetic variants. It aggregates multi-trait/single-trait association summary statistics across phenotypes while explicitly accounting for their correlation structure. The dimension of the phenotypes, or features (128), was much smaller than the sample size (N = 30,789). In this setting, a class of correlation-aware, summary-statistics-based multi-trait tests has been proven a standard and statistically valid framework in genetic epidemiology, with well-understood properties under the null.<br><br>Importantly, we assessed robustness and practical validity by benchmarking JAGWAS against widely used multi-phenotype GWAS approaches, including multi-ABEL and MOSTest. As documented in the JAGWAS manuscript, we observed high concordance in the identified loci and overall signal patterns across these methods. This consistency provides reassurance that the loci discovered by JAGWAS are unlikely to be artifacts driven by a flawed MANOVA model in the high-dimensional setting, where the

features have a dimension that exceeds the sample size. In our analysis, the number of features was much smaller than the sample size. To further evaluate the robustness of the JAGWAS findings, we additionally applied MOSTest to the 128 UDIP-FA phenotypes. the Q–Q plots showed that the P-value distributions from JAGWAS and MOSTest were highly concordant (Figure R2), supporting the reliability of the JAGWAS results.

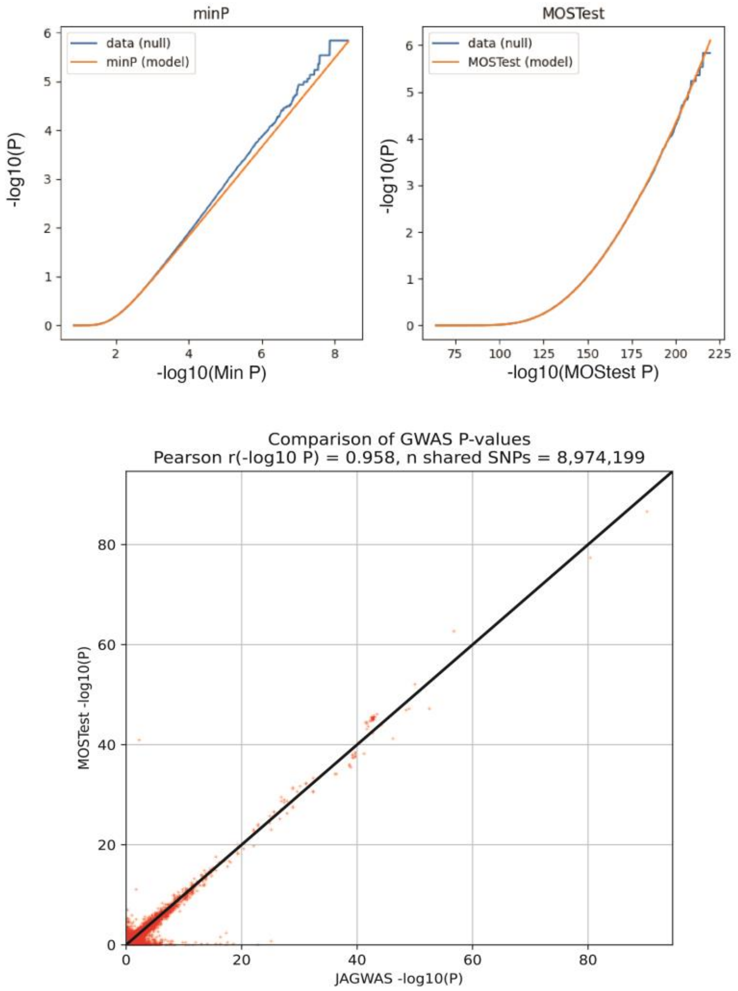

Figure R2 | Calibration and validation by the MOSTest.

(Top) Goodness-of-fit plots for the null distributions of minP (left) and MOSTest (right) statistics. The empirical null distributions derived from permutations (blue step lines) are compared against their respective theoretical models (orange curves). The close alignment indicates that the theoretical models are well-calibrated. (Bottom) Comparison of GWAS summary statistics between JAGWAS and MOSTest. The scatter plot displays the negative log-transformed P-values for 8,974,199 shared SNPs using JAGWAS (x-axis) and MOSTest (y-axis). The strong correlation (Pearson r = 0.958) along the diagonal demonstrates high concordance between the two methods.

Excerpt 1.3 (main text)	Not applicable.

REF1.4 – Explanation of UDIP

Referee Comment	*Minor Comments:*<br>*What exactly is the acronym UDIP stands for? It showed up very early in the abstract and introduction, without explicitly spelling it out first.*
Author Response	Thanks for your comments. UDIP stands for "Unsupervised Deep learning derived Imaging Phenotypes", referring to the latent representations derived from our unsupervised deep autoencoder model. In the revised manuscript, we now spell out the full term at its first occurrence in the Abstract and have checked the text to ensure consistent and unambiguous usage throughout.
Excerpt 1.4 (main text)	Line 16-18<br>"…it adopted the voxel-wise FA maps as the input, and to extract brain-wide Unsupervised Deep learning derived Imaging Phenotypes (UDIP) of FA maps—referred to as UDIP-FA"

REF1.5 – Issue of UMAP

Referee Comment	*Minor Comments:*<br>*It is unclear what UMAP can provide given that their 128 UDIP dimensions are relatively independent to each other.*
Author Response	Thank you for your comments. The UMAP only show that the UDIP-FA can represent the distribution of the FA values of WM tracts in individual level. and the supplementary figures (Figure S2, Figure R1) was showing the correlation within different dimensions of 128 UDIPs at phenotype level and genotype level.
Excerpt 1.5 (main text)	Not applicable

REF2 – Point-to-Point response to REF2

REF2.1 – Biological Interpretation of UDIP-FA

Referee Comment	*1) Biological Interpretation:*<br>*While the paper attempts to interpret the high-dimensional UDIP-FA features using the PerDI approach and regional enrichment, the complex, distributed patterns captured by the 128 dimensions remain a challenge for full, intuitive biological interpretation compared to simpler, atlas-defined measures.*<br>*I found the R2 values are reported for the 48 major tracts, and the UDIP-FA enrichment patterns across different WM tracts for dimension 1 and dimension 2 (UMAP embedding). In addition, some select UDIP-FA dimensions are described in lines 145-154. However, I do not seem to find enrichments for the 128 UDIP-FA features in the main text or supplements. Connecting the major WM tracts to these 128 UDIP-FA features could add some biological interpretation to each of these features, for example, UDIP-FA feature x could show enrichment like UMAP dimension 1 or 2, or could be widespread. In addition, such enrichment or something on those lines could be a stepping stone for the adoption of these high-dimensional UDIP-FA features by the readers working with the major WM tracts.*
Author Response	Thanks for your comments. We fully agree that connecting the 128 UDIP-FA features to major WM tracts is crucial for biological interpretation and will facilitate the adoption of these high-dimensional features.<br><br>We have analyzed the enrichment patterns for all 128 UDIP-FA features. However, presenting the comprehensive enrichment results for every dimension across all tracts resulted in a large volume of tabular data that is difficult to accommodate within the main text or standard supplementary files. To address this and ensure the data is user-friendly, we have hosted these detailed results on a dedicated companion website (https://udip-fa.github.io/Dim_visualization/).<br><br>While this link was previously listed in the Data Availability section, we agree that it is central to the interpretation of the results. Therefore, we have now explicitly added a reference to this website in the Results section to ensure readers can easily access and explore the specific tract-wise enrichments for each of the 128 features.
Excerpt 2.1 (main text)	Line 128-130<br><br>"We found that UDIPs were significantly more enriched in WM compared with GM and CSF (Figure 2b, Figure S5), and exhibited distinct enrichment patterns across different WM tracts (https://udip-fa.github.io/Dim_visualization/)."

REF2.2 – Interpretability of the diversity of UDIP-FA

Referee Comment	*Major Comments:*<br><br>*2) FA features:*<br>*For me, it seems that the high-dimensional UDIP-FA features are a mix of white matter integrity, brain morphology, and anthropometric measures. While overlap is expected, it's the ranking of overlap that seems to indicate that UDIP-FA's may be capturing other features, above white matter microstructure.*<br><br>*a) In the section "Comparison of UDIPs across different brain imaging modalities320", Lines 332-333: "Furthermore, loci identified across different models revealed a consistent pattern: T1 and FA shared 374 loci (80.09% overlap), while T2 shared 399 loci (86.18%) with the same set." This suggests UDIP-FA features capture a strong brain morphometric component.*<br><br>*b) In the section "Comparison with previous GWAS studies", in table S7, "Overlap of UDIP-FA–associated SNPs with the GWAS Catalog", shows anthropometric measures (e.g., Height, Waist circumference adjusted for body mass index, etc.) and brain morphology (e.g., Cortical surface area, Brain morphology (MOSTest), Cortical thickness, etc.) with most occurrences. The white matter trait that appears most is "White matter microstructure (radial diusivities)", with 118 occurrences. While indirect and dependent on the GWAS Catalog count, this seems to point to UDIP-FA may be capturing beyond FA features, including other DTI-derived measures.*<br>*It will help the readers if this could be quantified. For example, using the approach mentioned in lines 106-108: "Specifically, we extracted average FA values from 48 major white matter tracts defined by the UK Biobank and used multiple linear regression to assess the extent to which the 128 UDIP-FA features explained variance in each tract (Methods)." Here, the comparative measures could be global measures including FA, MD, RD, total SA, cortical thickness, ICV, BMI, and height, and one can assess the extent to which the 128 UDIP-FA features explain variance in such metrics.*<br><br>*In a scenario where these UDIP-FA go beyond FA, appropriate changes in the terminology and other parts of the paper may be warranted.*

Author Response	Thanks for your comments. First, we want to clarify that the cross-modal overlap we observed should not be surprising given the extensive shared genetic architecture reported across structural and diffusion MRI phenotypes in large-scale UK Biobank studies[2]. Recent multivariate work further demonstrates abundant pleiotropy across neuroimaging modalities, providing a broader context for why an FA-derived representation can show meaningful shared signals with T1/T2-based morphology and anthropometric traits[3].<br><br>Second, we would like to clarify that UDIP-FA is trained exclusively on FA maps and does not use T1/T2 images or anthropometric measures as input. Thus, by construction, UDIP-FA is an FA-derived representation of white-matter microstructure. The observed overlap with T1/T2-based loci and anthropometric traits therefore reflects shared biological and genetic influences across these modalities, rather than UDIP-FA directly "encoding" non-FA inputs.<br>In addition, following the reviewer's suggestion, we have now explicitly quantified how strongly UDIP-FA can account for variance in global imaging-derived and anthropometric measures. Specifically, we used multivariant regression (CCA) with the 128 UDIP-FA features as predictors and estimated the proportion of variance explained in six sets of outcomes: (1) white-matter tract–averaged radial diffusivity (RD), (2) mean diffusivity (MD), (3) total cortical surface area, (4) total cortical volume, (5) mean cortical thickness and (6) BMI. As summarized in Figure S4, UDIP-FA explains the largest proportion of variance in DTI-derived white-matter microstructure measures (WM tract RD and MD), a moderate proportion of variance in T1-based cortical morphology measures (surface area and volume, followed by cortical thickness), and a smaller yet detectable proportion of variance in BMI. This pattern indicates that UDIP-FA primarily reflects FA-related white-matter microstructure, while also capturing cross-modal variation that is shared with global brain morphology.<br>In line with this, we have revised the text throughout the manuscript to more clearly describe UDIP-FA as an FA-derived, data-driven representation of white-matter microstructure that also encodes shared variance with brain morphology and anthropometric traits, rather than as a purely "FA-only" measure. We also softened the wording in the section "Comparison of UDIPs across different brain imaging modalities" to emphasize that the observed locus overlaps across UDIP-T1, UDIP-T2 and UDIP-FA reflects the known shared genetic architecture of brain structure across modalities, rather than indicating that UDIP-FA is dominated by T1-like or anthropometric signals. We hope that these additional analyses and clarifications make the nature of the UDIP-FA representation clearer to readers.

	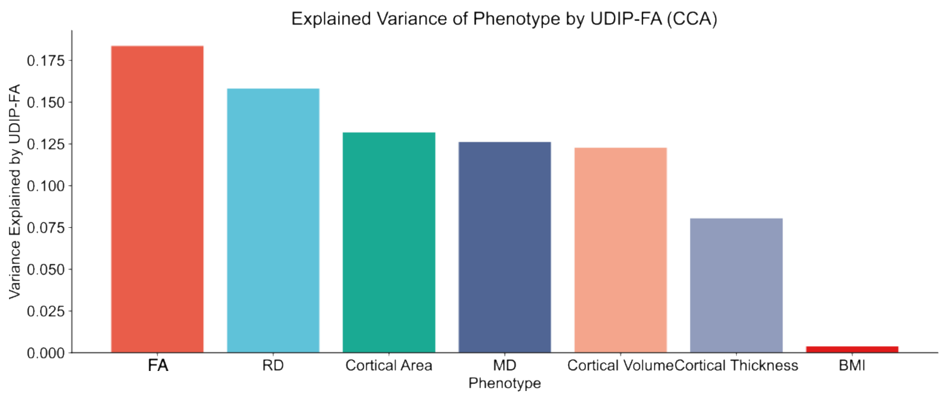<br><br>**Figure S4 | Shared variance between UDIP-FA and global structural and anthropometric phenotypes**. Proportion of variance in the 128 UDIP-FA features explained by different sets of imaging-derived and anthropometric measures, estimated using canonical correlation analysis (CCA). Feature sets include white-matter tract–averaged fractional anisotropy (FA), radial diffusivity (RD), mean diffusivity (MD), cortical surface area, cortical volume, mean cortical thickness and body mass index (BMI).
Excerpt 2.2 (main text)	Line 120-124<br>"To further validate the biological specificity of UDIP-FA, we quantified the variance explained by these features across diverse phenotypes. As expected, UDIP-FA explained the substantial variance in white matter microstructural metrics (tract-averaged FA and RD), significantly outperforming its predictive power for cortical morphology (e.g., surface area, volume) and anthropometric traits (e.g., BMI) (Figure S4)."<br>Line 441-445<br>"While UDIP-FA shares genetic loci with T1 and T2 modalities, this pattern likely reflects the extensive genetic pleiotropy that governs global brain development, where structural and microstructural traits naturally co-vary. At the same time, our phenotypic analyses demonstrate that UDIP-FA remains most strongly anchored to white matter microstructure, indicating clear modality-specific sensitive"<br>Line 715-717<br>"Additionally, we used multivariate regression to estimate the proportion of variance explained by the 128 UDIP-FA features in global white matter metrics (FA, RD, MD), cortical morphology (volume, area, thickness), and BMI."

REF2.3 – Predictive power of UDIP-FA

Referee Comment	*Major Comments:*<br><br>Predictive power:<br>In Lines 132-134, the authors mention: "UDIP-FA demonstrated strong sensitivity to age (mean absolute error (MAE) = 3.93 133 ± 0.04, Pearson's r = 0.77 ± 0.01; Figure 2c), outperforming a previously published study that used the traditional FA value from different WM regions[15]."<br>Since the training and test sample sizes could play a role in model evaluation, it would be appropriate if a baseline age prediction model using traditional FA value from different WM regions is used on the same training and test set.
Author Response	Thanks for your comments. We compared the performance of age and gender prediction between UDIP-FA features and traditional white-matter tract measures using cross-validation (Figure S7). The results show that UDIP-FA achieves substantially better predictive accuracy for both age and gender than traditional WM tract metrics.<br><br>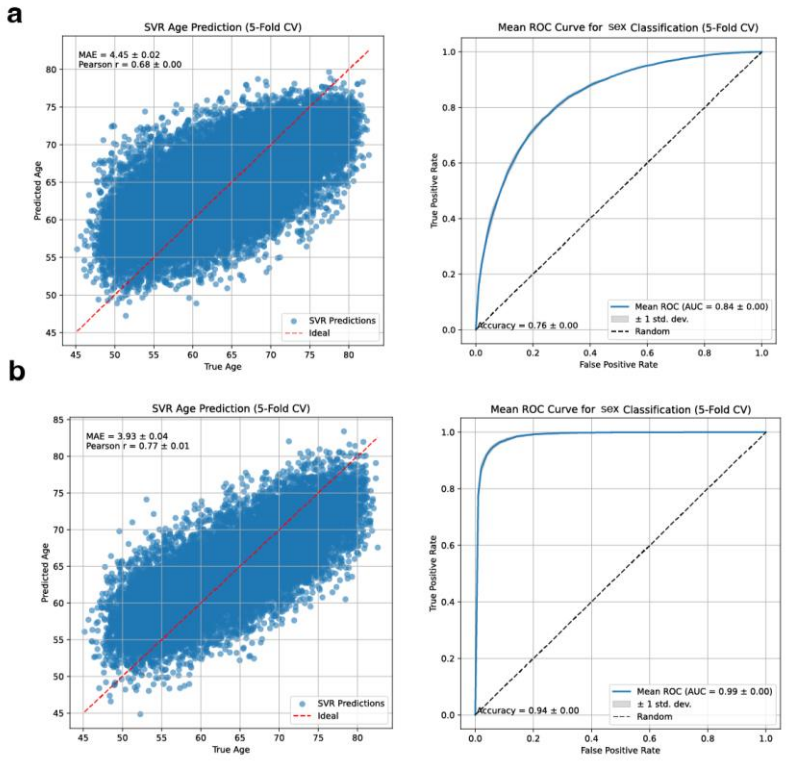<br><br>**Figure S7 | Comparison of prediction performance between traditional WM tracts (IDPs) and UDIP-FA features**. (a) Prediction performance using traditional white-matter tract IDPs. Left: Support Vector Regression (SVR) results for age prediction under 5-fold cross-validation, showing the relationship between predicted and true age (MAE = 4.45 ± 0.02; Pearson's r = 0.68 ± 0.00). Right: Mean ROC curve for sex classification across 5 folds (AUC = 0.84 ± 0.00; accuracy = 0.76 ± 0.00), with shaded regions

	indicating ±1 s.d. (b) Prediction performance using UDIP-FA features. Left: SVR-based age prediction under 5-fold cross-validation demonstrates improved accuracy relative to traditional IDPs (MAE = 3.93 ± 0.04; Pearson's r = 0.77 ± 0.01). Right: Sex classification ROC curve showing substantially enhanced discriminative ability (AUC = 0.99 ± 0.00; accuracy = 0.94 ± 0.00). Together, these results indicate that UDIP-FA features provide markedly stronger predictive performance than conventional WM tract measures.
Excerpt 2.3 (main text)	Line 145-147<br>"UDIP-FA demonstrated strong sensitivity to age (mean absolute error (MAE) = 3.93 ± 0.04, Pearson's *r* = 0.77 ± 0.01; Figure 2c), outperforming that used the traditional FA value from different WM regions (Figure S7)"

REF2.4 – Heritability comparison between UDIP-FA and WM features

Referee Comment	*Major Comments:*<br>*4) Heritability comparison:*<br>*In Lines 163-168, authors mention: "A total of 115 UDIP-FAs (89.8%) were significantly heritable (FDR-corrected P <0.05), with heritability estimates ranging from 6.9% to 85.2% (mean = 52.0%; Figure 3A and Table S2, (30.5%) UDIP-FAs showed high heritability (h2 > 0.6). The heritability of our UDIP-FAs was significantly higher than that of a previous study (mean h2 = 34.9%, P < 2.2e-16, Mann–Whitney U test, Figure 3b). This is remarkable as our UDIP-FAs are largely uncorrelated."*<br><br>*While the statistics seem correct.*<br>*I have a simple question: since these 128 UDIP-FA can be seen as a dimensionality reduction of the input FA maps and are largely uncorrelated, would an apple-to-apple comparison of heritability estimates involve comparing the heritability estimates derived from (for example) the top N principal components (>90% variance) across the WM tracts or input measures?*
Author Response	Thanks for your comments. We agree that comparing UDIP-FAs with Principal Components (PCs) provides a more rigorous "apple-to-apple" assessment of heritability for dimensionality-reduced features.<br><br>Following your recommendation, we extracted the top 24 PCs (explaining >90% of the variance) from the WM tracts and Top 905(explaining >90% of the variance) from the voxel-wise FA map, then calculated their heritability. As shown in the new Figure S9, the result

	confirms that UDIP-FAs capture highly heritable phenotypic information as effectively as standard dimensionality reduction techniques like PCA. We have included this comparison in the revised manuscript.<br>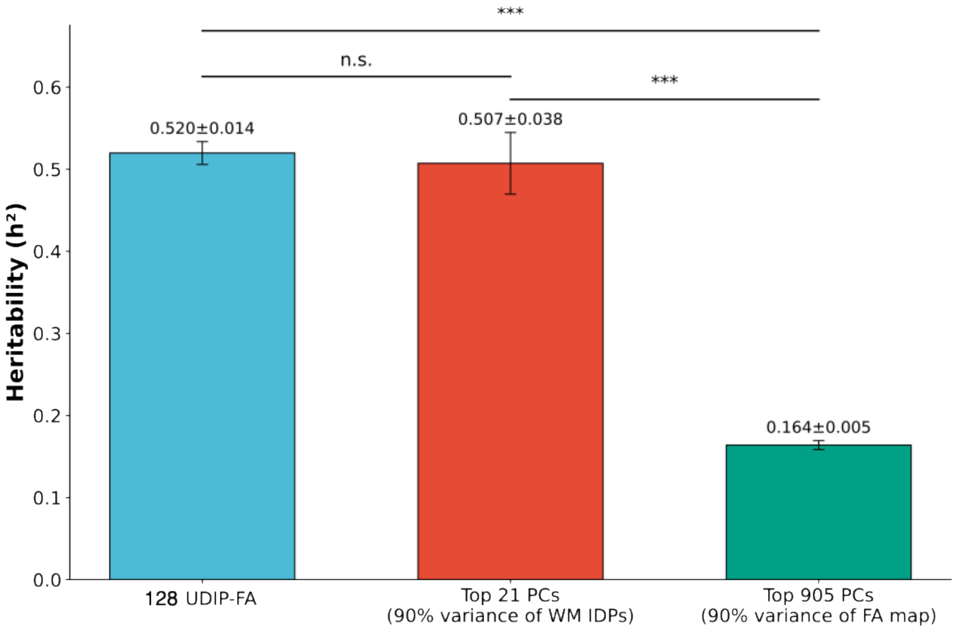<br><br>**Figure S9 | Comparison of SNP heritability across FA feature representations**. Bar plot showing the estimated SNP heritability (h^2) for three alternative representations of white-matter FA: all 128 UDIP-FA latent features, the top 21 principal components (PCs) explaining 90% of the variance of white-matter IDPs, and the top 905 PCs explaining 90% of the variance of the whole-brain voxelwise FA map. Bars indicate point estimates of h^2 with standard error, and numerical values above each bar denote the estimate ± s.e. Horizontal brackets summarize pairwise statistical comparisons (***P < 0.001; n.s., not significant).
Excerpt 2.4 (main text)	Line 182-185<br>"UDIP-FAs showed high heritability ($h^2 > 0.6$). The heritability of our UDIP-FAs was significantly higher than that of a previous study[12] (mean h^2 = 34.9%, P < 2.2e-16, Mann–Whitney U test, Figure 3b), and was higher than that of the top principal components (PCs) derived from WM tracts and voxel-wise FA maps (explaining >90% variance; Figure S9)."

REF2.5 – Interpreting the gene-based analysis

Referee Comment	*Major Comments:*<br>5) Interpreting the Gene-based analysis:

	While remarkable, the sheer number of "3,480" UFAGs seems too high to draw any specific conclusions.<br>For example, in lines 309-310: "This analysis revealed that 44 UFAGs interact with 87 drugs used to treat 55 distinct brain-related diseases (Table S15)". While specific examples are highlighted, could the authors guide the potential readers on addressing potential false positives?<br><br>While multivariate analysis of multiple measures may enhance the discovery of genetic variants, including MOSTest, van der Meer Nature Comm 2020, follow-up on such a large set creates another set of challenges. As such, providing a guide to the reader with not only specific examples, but also navigating false positives, would help the adoption of UDIP-FA.
Author Response	We thank the reviewer for this constructive comment. We agree that the identification of 3,480 UFAGs, while demonstrating the statistical power of the multivariate UDIP-FA framework, presents a challenge for interpretation. To address the concern regarding false positives and to provide a clear "guide" for readers to navigate this extensive gene list, we have revised the Discussion and Methods section. We now explicitly outline a prioritization framework that leverages the multi-layered evidence already present in our study to filter candidates from "statistical association" to "biological causality" and "clinical utility."<br>(1) **Addressing false positives through statistical rigor**: First, we emphasize that the high number of genes reflects the polygenic nature of white matter microstructure rather than statistical inflation. To control for false positives, our study employed a rigorous two-stage design (Discovery and Replication) followed by a meta-analysis. Only genes passing strict Bonferroni correction in the gene-based MAGMA analysis ($P < 0.05 / 19,294$) were retained. And for the functional mapping gene, all gene are passed the Bonferroni correction.<br>(2) **Guide for reader prioritization**. For the gene annotation from MAGMA, readers can utilize the MAGMA enrichment P-value rank (e.g., Top 5%) to identify the strongest statistical associations. In addition, we also provide three specific biological filters derived from our results. **a)** Evolutionary constraint (pLI Score). We advise readers to prioritize the subset of 686 genes with high probability of loss-of-function intolerance (pLI > 0.9). As noted in the manuscript, this subset represents genes under strong evolutionary constraint, reducing the likelihood of them being non-functional bystanders. **b)** Cell-type specificity. For the post analysis of UFAG**,** readers focusing on mechanisms should prioritize UFAGs enriched in glial cells (astrocytes and oligodendrocytes), as these show the strongest

	biological convergence with white matter integrity. **c)** Translational potential (The "High-Confidence" Set). We have highlighted the 44 UFAGs interacting with 87 drugs (Table S15) as the set for translational research. Specific examples, such as ACHE (Alzheimer's treatment target) and ALDH2, serve as proof-of-concept for this prioritization strategy. For other interactions from Table S15, reader can use the rank of the interaction score to choose the high confidence paired between UFAG and drugs of brain disorders.
Excerpt 2.5 (main text)	Line 419-427<br>"While these specific examples illustrate the mechanistic insights from our findings, we acknowledge that navigating the full set of 3,480 UFAGs presents a challenge. To assist readers in interpreting these results, we suggest a potential prioritization strategy based on the multi-layered evidence in our study. For instance, candidates exhibiting high evolutionary constraint (pLI > 0.9) or specific enrichment in glial cells (astrocytes and oligodendrocytes) could be prioritized for mechanistic investigation. Similarly, for translational research, the 44 UFAGs interacting with known drugs (Table S15), such as the ANGPT1 may serve as a high confidence starting point. This perspective offers a possible roadmap for extracting biologically and clinically relevant targets from the broader statistical associations."

REF2.6 – Visualization of UMAP

Referee Comment	*Minor Comments:*<br>*1) Lines 103-105: "As shown in Figure 2a, UDIP-FA representations captured meaningful population-level variation, with FA values exhibiting a smooth gradient across the latent space." This gradient is not as standout as Fig 2 in ref [13], Patel et al, 2024, and the gradient only seems to be along the x-axis. If it helps, could you please explore the possibility of marginal density/histogram plots to illustrate the gradient point?*
Author Response	Thanks for your comments. In the revised manuscript, we have substantially updated Figure 2 to enhance the visibility of the FA gradient. We improved the color mapping and transparency and added marginal density plots to visualize the distribution shifts as suggested. Notably, we replaced the original Figure 2a with the embedding color-coded by the Fornix FA values. We selected the Fornix for the main figure as it exhibits a particularly distinct

<table>
<tr>
<td></td>
<td>

and continuous gradient, providing a clearer demonstration of how UDIP-FA accurately describes population-level microstructural variation.

To confirm that this structural encoding is consistent across the brain, we have also included supplementary figures displaying the latent space representations for other major white-matter tracts (e.g., corpus callosum). These additional visualizations further validate that UDIP-FA captures anatomically meaningful and smoothly varying white-matter structure across diverse anatomical regions

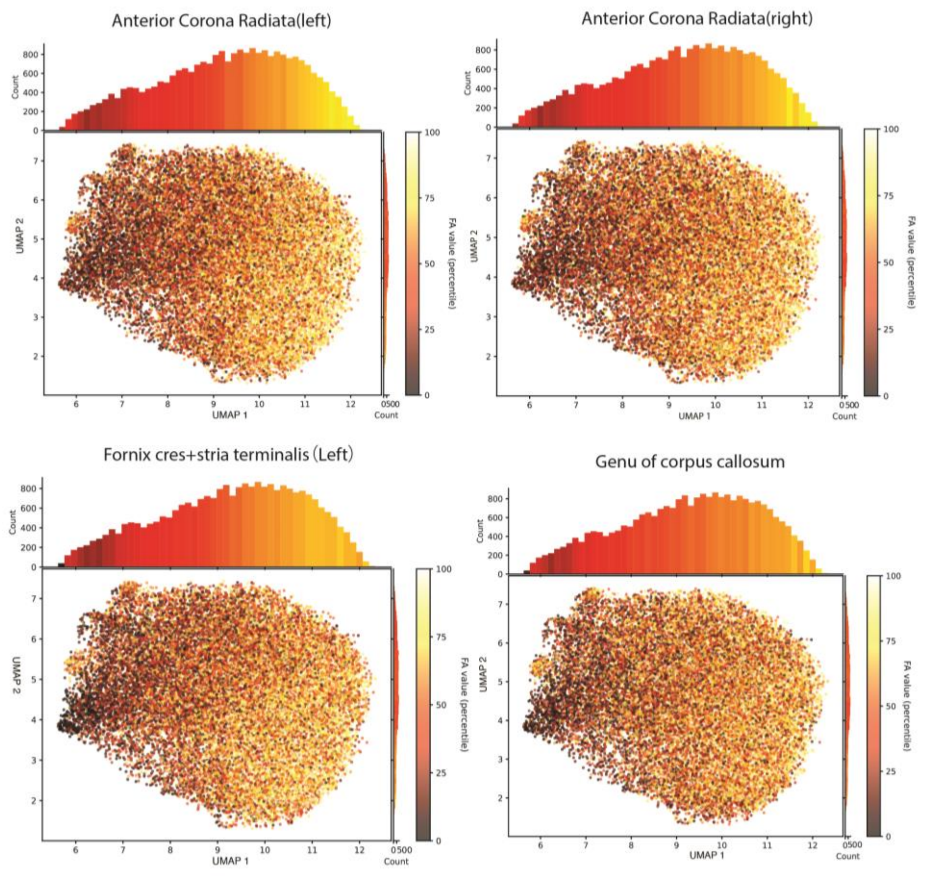

Figure S3 | UMAP visualization of tract-specific FA variation across the UDIP-FA latent space. Two-dimensional UMAP embeddings of the 128-dimensional UDIP-FA representations are shown for all participants. Each point denotes one individual, and points are colored by the individual's mean fractional anisotropy (FA) within the indicated white-matter tract, expressed as a percentile across the cohort (color bar). Marginal histograms (top and right) depict the sample density along UMAP1 and UMAP2, respectively. Panels correspond to the left and right anterior corona radiata, the left fornix cres+stria terminalis, and the genu of the corpus callosum.

</td>
</tr>
<tr>
<td>Excerpt 2.6 (main text)</td>
<td>Not applicable.</td>
</tr>
</table>

REF2.7 – Samples of brain diseases

Referee Comment	*Minor Comments:*<br>Lines 135-138: "In addition, we evaluated the ability of UDIP-FA to predict the status of six brain disorders: Alzheimer's Disease (AD), Depression (DEP), Epilepsy (EPI), Multiple Sclerosis (MS), Parkinson's Disease (PD), and Schizophrenia (SCZ). Patients with these conditions were identified from the UK Biobank (UKB) dataset using ICD-10 diagnostic codes (field 41270, Methods)." The sample size (case-control) information is only provided for MS cases on line 521. Please add the same for other disorders.
Author Response	Thanks for your comments. We have modified the manuscript and add the sample size of each disorder.
Excerpt 2.7 (main text)	Line 552-555<br>"To get the disease status of the UKB samples, we used the ICD-10 code (field: 41270) to obtain the patients who have the brain image data for different brain disorders, including AD (F00, G30, N=63), PD (G20, N=102), SCZ(F20, N=36), DEP(F32, N=2550), EPI (G40, N = 416), BIP(F31, N=128), and MS(G35, N=216)."

REF2.8 – Category of disorders

Referee Comment	*Minor Comments:*<br>Terminology for disorders. If I may suggest, please use neurological and psychiatric disorders for neurological (AD, PD, etc.) and psychiatric disorders (behaviourally defined, SCZ, DEP, etc.). For example, line 203: "for several neurological disorders, including schizophrenia (SCZ)33"
Author Response	Thanks for your comments. In the revised manuscript, we now consistently distinguish between neurological disorders (e.g., Alzheimer's disease, Parkinson's disease) and psychiatric disorders (defined, e.g., schizophrenia, depression) throughout the text. Specifically, the sentence at line 203 has been corrected to refer to "psychiatric disorders, including schizophrenia (SCZ)…" rather than grouping SCZ under neurological disorders.
Excerpt 2.8 (main text)	Line 139-140:<br>UDIP-FA demonstrates predictive power for age, sex, and neurological and psychiatric disorders

	Line 149-151<br>"…categorized into neurological (Alzheimer's Disease (AD), Epilepsy (EPI), Multiple Sclerosis (MS), Parkinson's Disease (PD)) and psychiatric disorders (Depression (DEP), Schizophrenia (SCZ)).<br>Line 552-555:<br>"To get the disease status of the UKB samples, we used the ICD-10 code (field: 41270) to obtain the patients who have the brain image data for different brain disorders, including neurological disorders(AD (F00, G30, N=63), PD (G20, N=102), EPI (G40, N = 416), MS(G35, N=216)), and psychiatric(SCZ(F20, N=36), DEP(F32, N=2550))."

REF2.9 – Baseline model of WM tract

Referee Comment	*Minor Comments:*<br>In the section "UDIP-FA demonstrates predictive power for age, sex, and neurological disorders", the baseline for comparison could be a standard model with major WM tract measures. Since the case and control groups are imbalanced, and training involves sampling an equal number of cases, it would be more appropriate to provide a baseline model for comparison.
Author Response	Thanks for your comments. We agree that establishing a baseline model is essential to rigorously evaluate the predictive power of UDIP-FA, particularly given the class imbalance and the sampling strategy employed.

	To address this, we have implemented a baseline model using standard measures from major white matter (WM) tracts that. Specifically, we extracted mean Fractional Anisotropy (FA) values from 48 standard tracts (based on the JHU ICBM-DTI-81 atlas) and trained a LightGBM classifier using the same cross-validation and down-sampling strategy as used for UDIP-FA. As illustrated in the Figure S7, UDIP-FA demonstrates robust predictive power across multiple disorders. notably, it achieves higher AUC scores compared to the baseline in Schizophrenia and Epilepsy. This indicates that the latent representations learned by UDIP-FA successfully capture complex, non-linear pathological patterns that are not fully represented by traditional tract-averaged FA values. While standard tract measures remain effective for diseases with gross white matter damage (e.g., MS), UDIP-FA offers a significant advantage in detecting the more subtle microstructural anomalies associated with psychiatric and neurological disorders like SCZ and EPI. Overall, this comparison demonstrates that UDIP-FA provides complementary and often superior predictive value, particularly for disorders with subtle or diffuse white matter pathology.<br>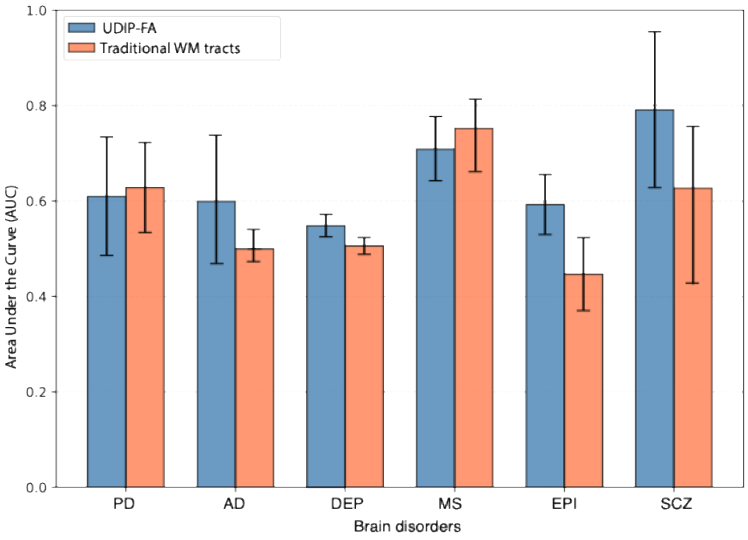<br><br>**Figure S8 ｜Comparative predictive performance of UDIP-FA and traditional white matter tract measures across neurological and psychiatric disorders.** The bar chart displays the Area Under the Curve (AUC) scores for the classification of six distinct brain disorders. The proposed UDIP-FA model (blue) is compared against a baseline model (orange) trained on mean fractional anisotropy (FA) values extracted from major white matter tracts. Data are presented as the mean AUC across cross-validation folds, with error bars representing the standard deviation. PD, Parkinson's disease; AD, Alzheimer's disease; DEP, depression; MS, multiple sclerosis; EPI, epilepsy; SCZ, schizophrenia.
Excerpt 2.9 (main text)	Line 154-160

	"To rigorously benchmark performance, we compared UDIP-FA against a baseline model derived from standard white matter tract measures (Figure S8). The results demonstrated that UDIP-FA effectively captures disease-specific signals, achieving an average classification performance with an AUC of 0.642 ± 0.082 (Figure 2d), and offering complementary predictive value to traditional tract-based metrics in most disorders. Furthermore, we analyzed the feature importance of the classification model to investigate potential disorder-specific white matter patterns"

REF2.10 – Interpretability of the UDIP-FA for SCZ

Referee Comment	*Minor Comments:*<br><br>In the section "UDIP-FA demonstrates predictive power for age, sex, and neurological disorders", the most prominent UDIP-FA features for AD, MS, and EPI are described. However, the exclusion of SCZ, the one with the highest AUC, seems perplexing. If the UDIP-FA features are more widespread for SCZ, that would be interesting. This difference could reflect the neurological and psychiatric disorders.
Author Response	Thanks for your comments. We initially did not highlight SCZ in the detailed feature discussion primarily due to the relatively smaller sample size of the SCZ cohort(N=36) compared to other disorders in our dataset. We exercised caution to avoid over-interpreting feature rankings that might be less stable due to limited data.<br><br>However, following your suggestion, we examined the top-contributing features for SCZ classification and found that Dimension 47 (Dim47, https://udip-fa.github.io/Dim_visualization/) exhibited high importance. This dimension is spatially enriched in the Uncinate Fasciculus (UNC) and the Inferior Fronto-Occipital Fasciculus (IFO). Existing literature suggests that SCZ is a disorder affecting widespread white matter tracts rather than being confined to isolated regions[1]. A key advantage of UDIP-FA is its ability to characterize FA patterns that span across multiple tracts (as captured by dim47). This capability allows our model to effectively capture the diffuse microstructural alterations typical of SCZ, which likely explains the high AUC performance.<br><br>We also wish to clarify that the primary goal of this classification task was to demonstrate UDIP-FA's capacity to capture disease-relevant biological information, rather than to advocate for its immediate use as a clinical tool

	for disease diagnosis. We have revised the Results and Discussion section to incorporate these insights regarding SCZ and to explicitly state the scope of this analysis to prevent misunderstanding.
Excerpt 2.10 (main text)	Line 148-160<br><br>"To evaluate the capacity of UDIP-FA to characterize pathological white matter patterns, we assessed its association with six major brain disorders, categorized into neurological (Alzheimer's Disease [AD], Epilepsy [EPI], Multiple Sclerosis [MS], Parkinson's Disease [PD]) and psychiatric disorders (Depression [DEP], Schizophrenia [SCZ]). Patients were identified from the UK Biobank (UKB) dataset using ICD-10 diagnostic codes (field 41270, Methods). We trained a gradient-boosted decision tree (GBDT) classifier using a stratified cross-validation strategy with down-sampling to address class imbalance. To rigorously benchmark performance, we compared UDIP-FA against a baseline model derived from standard white matter tract measures (Figure S8). The results demonstrated that UDIP-FA effectively captures disease-specific signals, achieving an average classification performance with an AUC of 0.642 ± 0.082 (Figure 2d), and offering complementary predictive value to traditional tract-based metrics in most disorders. Furthermore, we analyzed the feature importance of the classification model to investigate potential disorder-specific white matter patterns."

REF2.11 – Gene list of ASD

Referee Comment	*Minor Comments:*<br><br>*The number of risk genes, Table S13, specifically for ASD, a staggering 968 genes, seems perplexing. See, for example, Wagstyl et al. eLife (2024) https://doi.org/10.7554/eLife.86933.3. Would the findings remain similar if curated disease gene sets from other published work were explored?*
Author Response	Thanks for your comments. We acknowledge that the number of risk genes in Table S13, derived from the comprehensive AutDB database (968 genes), is substantially larger than lists focusing on high-confidence variants (e.g., ~69 genes only focus on rare variants in Wagstyl et al. 2024). This difference reflects our initial intent to capture the broad polygenic architecture of UDIP-FA, which includes common variants beyond high-penetrance mutations. However, to address the concern regarding robustness and the potential for false positives in such a broad list, we performed a sensitivity analysis using the stricter SFARI Gene High Confidence (Category 1) dataset[4]. As shown in the Figure R3, the enrichment of UFAGs within this high-confidence set

<table>
<tr>
<td></td>
<td>

is not only significant but notably stronger than in the broader AutDB set (SFARI: $P = 2.46 \times 10^{-4}$, Odds Ratio = 1.75 vs. AutDB: $P = 0.068$, Odds Ratio = 1.14). This result confirms that our findings are not driven by "noise" in the larger list; rather, the biological link between UDIP-FA and ASD becomes even more pronounced when focusing on core pathogenic genes. In addition, we have modified the title of Table S13 to "Collection of candidate genes risk of 8 different brain disorders."

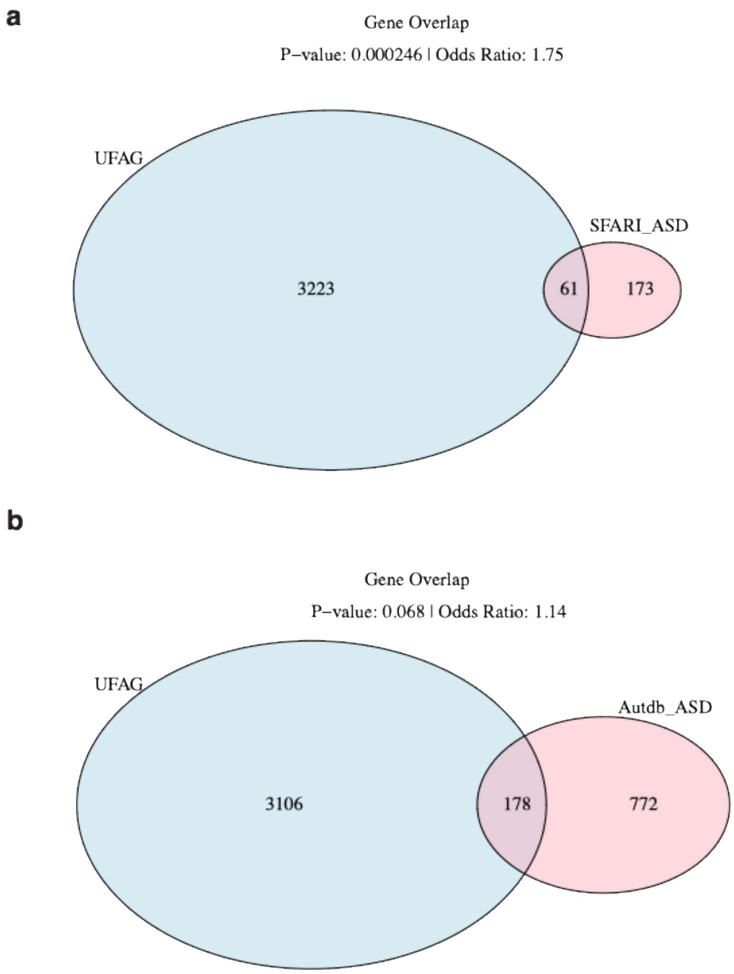

Figure R3 | Sensitivity analysis of UFAG enrichment in ASD risk gene sets. (a) Venn diagram illustrating the significant enrichment of UFAGs within the high-confidence ASD risk gene set (Category 1) from the SFARI Gene database. **(b)** Venn diagram showing the overlap between UFAGs and the comprehensive ASD risk gene set from the AutDB database. Significance was calculated using Fisher's exact test.

</td>
</tr>
<tr>
<td>Excerpt 2.11 (main text)</td>
<td>Not applicable</td>
</tr>
</table>

REF3 – Point-to-Point response to REF3

REF3.1 – Global summary

Referee Comment	*Overall, this is a solid and well-executed study. The methodology is clearly described, the analyses are comprehensive, and the results are biologically plausible and internally consistent. The manuscript convincingly demonstrates the potential of unsupervised deep representations for imaging-genetics research.*
Author Response	We sincerely thank the reviewer for this positive and encouraging assessment of our work.
Excerpt 2.1 (main text)	Not applicable

REF3.2 – Stability and reproducibility of embeddings

Referee Comment	*Major Comments:*<br>1) Stability and reproducibility of embeddings: Have the authors evaluated how stable the UDIP-FA latent space is across different random initializations, training runs, or subsets of data? Are the same latent dimensions consistently recovered, or is there variability between models?
Author Response	Thanks for your comments. We understand the concern that deep learning embeddings can sometimes be sensitive to stochastic factors like random initialization. To address this, we wanted to ensure our results were robust not just to random initialization, but to the model architecture itself. Therefore, we tested the stability of the UDIP-FA latent space by training independent models across a wide range of dimensions—from 32 to 1024—and conducted two systematic evaluations:<br>1. **Latent space consistency**. We computed the pairwise Canonical Correlation Analysis (CCA) between the features extracted from models with different dimensions (e.g., 32- dimension vs. 1024-dimension). As shown in the Figure S16a, the canonical correlations between different models are extremely high, consistently exceeding 0.99 (ranging from 0.997 to 1.000). This near-perfect alignment demonstrates that the core biological information captured by UDIP-FA is invariant to model capacity and initialization. Whether compressed to 32 dimensions or expanded to 1024, the model consistently recovers the same underlying phenotype

	manifold. This confirms that UDIP-FA is not a stochastic artifact but a stable representation of white matter microstructure.<br><br>2. **Robustness of downstream genetic discovery**. We then evaluated whether this stability translates to downstream applications by performing GWAS on the phenotypes derived from each dimension setting (32, 64, 128, 256, 512, 1024). As shown in Figure S16b, the number of identified significant SNPs is robust across dimensions. While higher dimensions (256 and 512) yield a slight increase in discovery power (peaking at 139 SNPs for 512-dim), the 128-dimensional model (used in our manuscript) captures most of the genetic signal (122 significant SNPs) while maintaining model parsimony. This sensitivity analysis justifies our choice of 128 dimensions as an optimal balance between complexity and statistical power, further reinforcing the reliability of our reported findings.<br><br>In summary, our additional experiments confirm that the UDIP-FA latent space is highly stable, reproducible, and robust to variations in model training configurations. We have included these results in the supplementary figures and updated the Methods section accordingly.

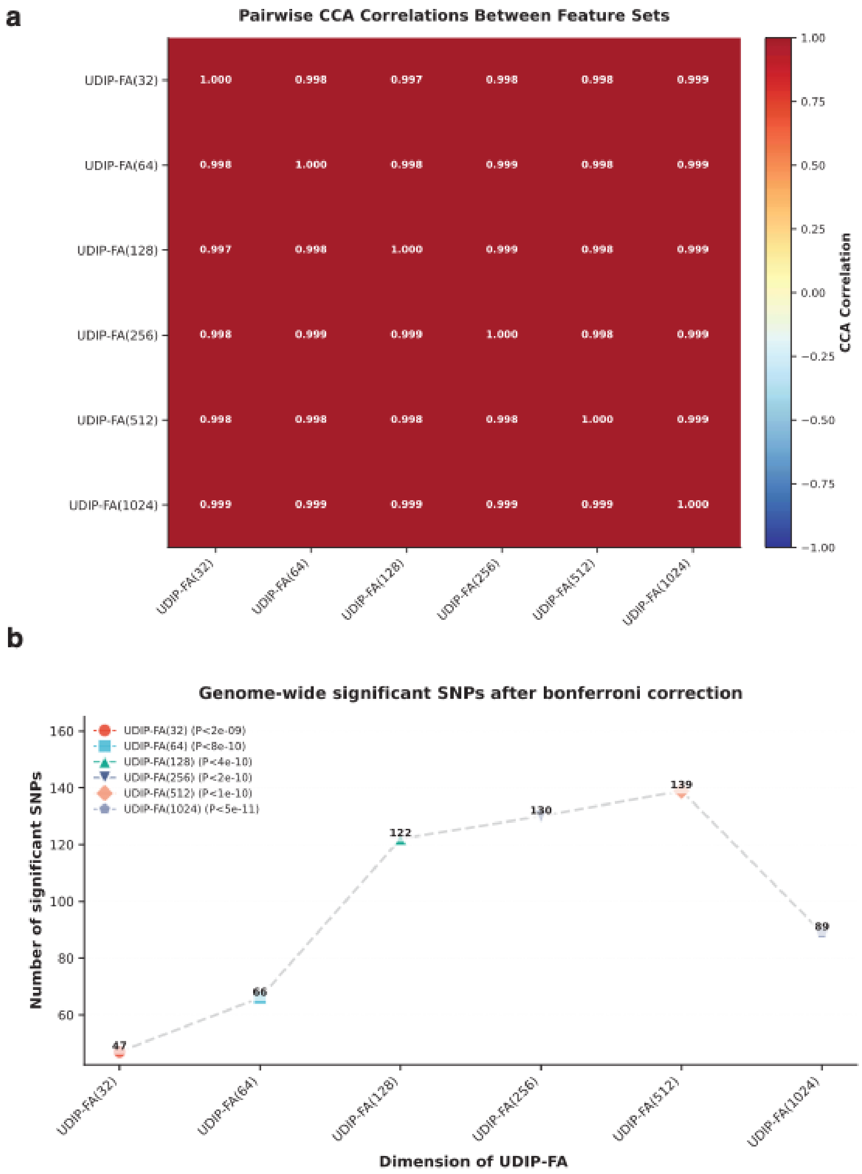

Figure S16 | Evaluation of UDIP-FA stability and model selection. (a) Pairwise Canonical Correlation Analysis (CCA) heatmap showing the similarity between UDIP-FA features extracted from models with different latent dimensions (32, 64, 128, 256, 512, and 1024). The extremely high correlation coefficients (>0.99) indicate that the latent space structure is highly stable and consistent regardless of the dimension size or random initialization. **(b)** Sensitivity analysis of genetic discovery power across different latent dimensions. The line plot shows the number of genome-wide significant SNPs identified (after Bonferroni correction) for each dimension setting. The 128-dimensional model (selected for this study) achieves robust discovery power comparable to higher-dimensional models, balancing information content with parsimony.

Excerpt 3.2 (main text)	Line 513-521<br><br>"To evaluate the stability and reproducibility of the UDIP-FA latent space against stochastic factors (e.g., random initialization) and model capacity, we trained independent models across a range of latent dimensions (32, 64, 128, 256, 512, and 1024). We assessed the consistency of the learned representations using pairwise Canonical Correlation Analysis (CCA) between models and evaluated the robustness of downstream genetic discovery by performing GWAS across all dimension settings. The results demonstrated high latent space consistency (CCA > 0.99) and robust genetic signal discovery across dimensions (Figure S16). Consequently, the 128-dimensional model was selected for the final analysis as it offered an optimal balance between model parsimony and statistical power."

REF3.3 – External validation of UDIP-FA

Referee Comment	*Major Comments:*<br><br>External validation: Can the pretrained model be applied to other cohorts or scanners? If so, how well do the embeddings generalize, and what procedures (if any) are needed to align new data to the UDIP-FA space?
Author Response	Thanks for your comments. In this work, reproducibility was primarily assessed at the genetic-association level within UK Biobank, using a discovery–replication design. Specifically, UDIP-FA features were derived once from the standardized UKB/FSL/MNI-aligned FA maps, and all downstream GWAS analyses were carried out in a discovery subset with independent replication in a hold-out sample. The high concordance of loci and enrichment patterns between discovery and replication provides initial evidence that the learned representations are reusable under consistent preprocessing and acquisition conditions.<br><br>At present, we do not yet have access to sufficiently large external diffusion MRI datasets processed under comparable pipelines to perform a full cross-cohort validation. Nevertheless, our public codebase already provides a ready-to-use interface for external users to project their own diffusion maps into the pretrained UDIP-FA space, compute reconstruction error, and visualize latent features (https://udip-fa.github.io). This enables independent evaluation of generalization as new data become available.<br><br>We have explicitly discussed this limitation in the Discussion section and view external validation as an important future step. Ongoing work in our group aims to apply the pretrained UDIP-FA model to other large-scale

	diffusion datasets such as ABCD to systematically assess cross-scanner and cross-protocol robustness.
Excerpt 3.3 (main text)	Line 454-459<br>"Furthermore, while our discovery-replication design confirmed internal reproducibility under standardized UK Biobank acquisition, we have not yet assessed the model's generalization to external cohorts with varying scanners or protocols. To facilitate community validation, we have released the projection interface (https://udip-fa.github.io), and future studies will aim to systematically validate the robustness of these findings across diverse populations and large-scale external datasets."

REF3.4 – Remarks on code availability

Referee Comment	*Comments:*<br>While the study provides extensive downstream analysis code, the repository currently lacks the full training and inference pipeline for the autoencoder model used to derive the UDIP-FA embeddings.<br><br>For reproducibility and broader utility, it would be important for the authors to release or document in detail the complete procedure—from preprocessing of raw FA maps to generation of UDIP-FA features—including model architecture, training parameters, and inference steps.<br><br>In addition, a public script or clear instructions to project new cohorts into the pretrained UDIP-FA space would enable independent validation and cross-cohort applications, which are essential for evaluating the robustness and generalizability of the proposed framework.
Author Response	Thank you for this helpful suggestion. We fully agree that making the full UDIP-FA pipeline transparent and easily reusable is critical for reproducibility and broader utility.<br>In the revised GitHub, we have substantially expanded the public GitHub repository to address these points:<br>1. End-to-end pipeline documentation.<br>We now provide a step-by-step description of the complete workflow from raw FA maps to UDIP-FA embeddings, including:<br>Detailed model architecture specification (encoder/decoder structure, latent dimensionality, normalization layers, etc.);

	Training setup and hyperparameters (loss components, learning rate schedule, batch size, number of epochs, optimizer, data augmentation, and checkpointing strategy);<br>Inference procedure for generating UDIP-FA features from trained checkpoints.<br>2. Scripts for training and inference.<br>We added example scripts/notebooks that can be run directly to: Train the UDIP-FA autoencoder from scratch on preprocessed FA data; Load a pretrained checkpoint and extract UDIP-FA embeddings for a given cohort.<br>3. Projection of new cohorts.<br>To facilitate independent validation and cross-cohort applications, we now include a dedicated script and instructions for projecting new cohorts into the pretrained UDIP-FA space.
Excerpt 3.4 (main text)	Not applicable.

<u>Reference</u>

[1] Kelly S, Jahanshad N, Zalesky A, Kochunov P, Agartz I, Alloza C, et al. Widespread white matter microstructural differences in schizophrenia across 4322 individuals: results from the ENIGMA Schizophrenia DTI Working Group. Mol Psychiatry 2018;23:1261-9.
[2] Elliott LT, Sharp K, Alfaro-Almagro F, Shi S, Miller KL, Douaud G, et al. Genome-wide association studies of brain imaging phenotypes in UK Biobank. Nature 2018;562:210-6.
[3] Tissink EP, Shadrin AA, van der Meer D, Parker N, Hindley G, Roelfs D, et al. Abundant pleiotropy across neuroimaging modalities identified through a multivariate genome-wide association study. Nat Commun 2024;15:2655.
[4] Banerjee-Basu S, Packer A. SFARI Gene: an evolving database for the autism research community. Dis Model Mech 2010;3:133-5.

Response Letter to

Unveiling genetic architecture of white matter microstructure through unsupervised deep representation learning of fractional anisotropy maps

REF1 – Point-to-Point response to REF1

REF1.1 – Comparison between UDIP-FA and PC

<table>
<tr><td>Referee Comment</td><td>Major Comments:

While Figure R1 is informative in a way by comparing to more traditional PC approach, it is a bit off target. The key metrics they used to justify UDIP is a). loci discovery, b). trait predictions, and c). genetic signal enrichment. However, none of those are compared head-to-head comparison.</td></tr>
<tr><td>Author Response</td><td>Thanks for your comments. We have revised the comparison to provide a clearer head-to-head evaluation between UDIP and the conventional PC based approach, organized around the three criteria that motivated UDIP.

First, to directly compare loci discovery between UDIP and PC, we applied the same minP-based aggregation strategy to the GWAS summary statistics from each representation, followed by locus definition and annotation using FUMA. Under the corresponding multiple-testing thresholds ($5 \times 10^{-8}/128$ for UDIP and $5 \times 10^{-8}/24$ for PC), UDIP yielded substantially more association signals than PCA as reflected by higher numbers of genomic risk loci, lead SNPs, independent significant SNPs, and mapped genes (Figure S17).

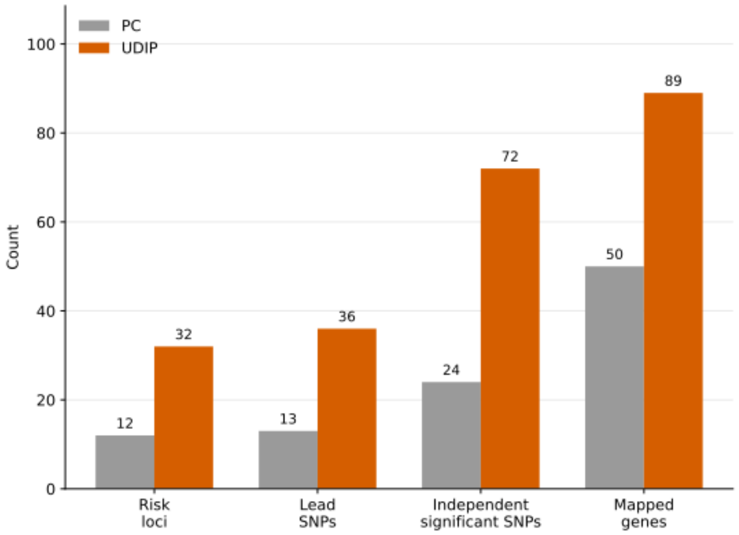

Fig. S17 | Direct head-to-head comparison of loci discovery between IDP-PC and UDIP.

Second, for trait prediction, we now include a direct comparison showing that UDIP-derived features provide stronger predictive performance than PCA-derived features for the traits evaluated (Figure S18).</td></tr>
</table>

<table>
<tr>
<td></td>
<td>
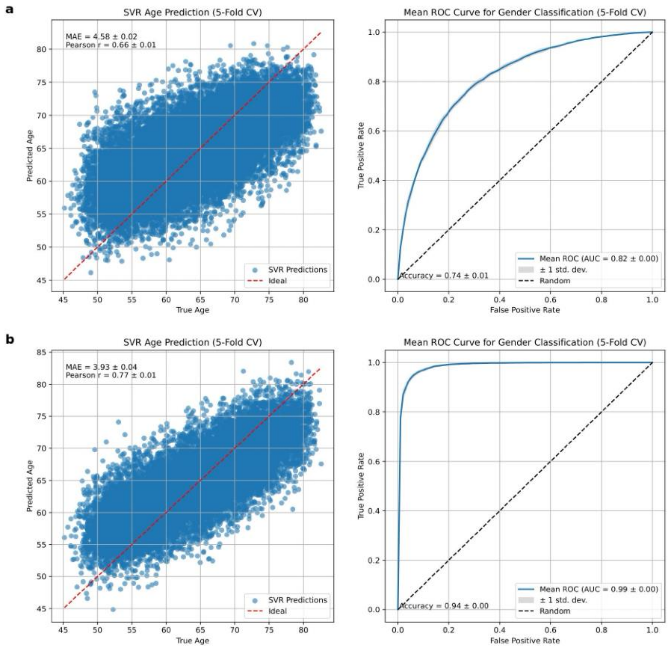

Figure S18 | head-to-head comparison of trait prediction performance using PC (a) versus UDIP (b) across the evaluated traits (age and sex).

For genetic signal enrichment, we revised the analysis into a direct head-to-head comparison focused on FA-relevant biology (**Fig.R3**). UDIP generally outperformed PC across most evaluated categories, although the magnitude of improvement varied across traits. Importantly, this advantage was also reflected in the summary panel, where UDIP showed both a higher overall overlap rate and a stronger overlap with GWAS Catalog traits related to diffusion MRI/white matter. Together, these results suggest that UDIP captures signals that are more consistently aligned with previously reported white-matter-related biology than PC.

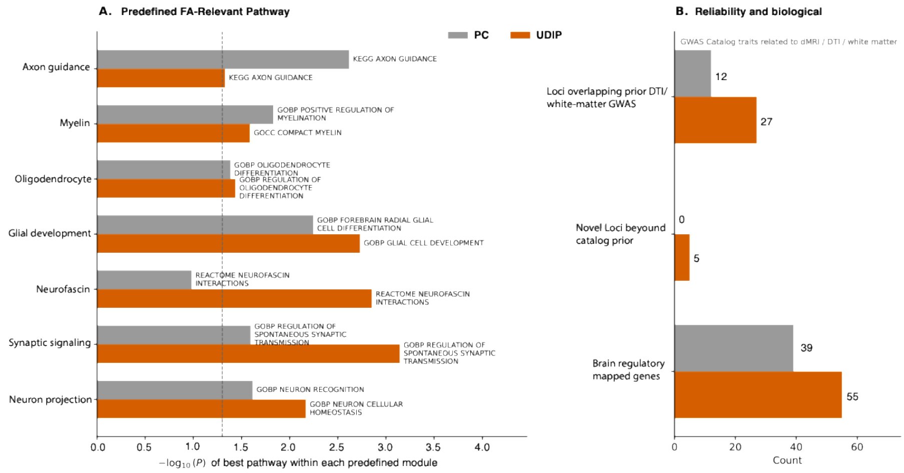

Figure S19 | Head-to-head comparison of PCA and UDIP in biological enrichment.
</td>
</tr>
<tr>
<td>Excerpt 1.1
(main text)</td>
<td>Line 349-358:</td>
</tr>
</table>

	" Compared with conventional FA phenotypes derived from hand-crafted ROI- or tract-based approaches and PCA-based representations, UDIP-FA is learned directly from whole-brain FA maps using an unsupervised 3D convolutional autoencoder. As demonstrated in our analyses, this framework preserves richer information on distributed white matter organization, including inter-regional relationships that are not fully retained by predefined anatomical summaries or linear dimensionality reduction methods. We used predictive modeling tasks and biological annotation analyses (Figures S16-S18), including brain age estimation and loci discovery, primarily as proof-of-concept evaluations of the biological informativeness of these features rather than as evidence for immediate clinical diagnostic utility, particularly given the limited sample sizes available for certain disorders. "

REF1.2 – Prediction of UDIP-FA

Referee Comment	*Major Comments:*<br>*Though I disagree on the interpretation on how AUC varies according to prevalence, I can accept the way authors revised the manuscript and tried matching schema to mitigate the unbalanced design.*
Author Response	Thank you for your comments.
Excerpt 1.2 (main text)	No applicable.

REF1.3 – Validity of JAGWAS

Referee Comment	Major Comments:<br>*3. It is good for authors to compare their JAGWAS to other methods such as MOSTest. However, the correlations between p-values let the skewed extreme cases dominate the evaluation while ignore those divergent cases in the P = E^-10~20 range (Figure R1). It still does not answer a critical question - does JAGWAS control type I error sufficiently given the significance threshold they imposed*?

Author Response	Thank you for your comments. To directly evaluate whether JAGWAS can sufficiently control type I error rates at the genome-wide significance level, we performed a large-scale simulation with 2 billion p-values generated under the null hypothesis of no genotype–phenotype associations with any of the 128 phenotypes. The per-SNP z-scores across the 128 traits were randomly drawn from a multivariate normal distribution, $z \sim MVN(0, R)$, where R is the cross-trait correlation matrix estimated from the real data after covariate adjustment. For each of 225 independent simulation replicates, we generated single-phenotype GWAS z-scores under the null for 8,925,936 SNPs across 128 traits. We then applied JAGWAS to these simulated z-scores to obtain multi-phenotype association test p-values. Each simulation replicates therefore produced 8,925,936 p-values, resulting in a total of 225 × 8,925,936 ≈ 2.008×10^9 null p-values. This scale was chosen to ensure stable estimation of Type I error in the extreme tail at the genome-wide significance level of 5×10^{-8}. This procedure directly evaluates the empirical Type I error rate of JAGWAS under the null hypothesis of no genotype-phenotype associations with any of the 128 phenotypes.<br><br>Under the null, p-values closely followed the expected uniform distribution on the interval [0, 1] (genomic inflation factor $\lambda_{GC} = 1.001$), with no evidence of systematic inflation (**Fig. R1**). Tail calibration across multiple thresholds further confirmed appropriate control of false positives. At the genome-wide significance threshold of 5×10^{-8}, we observed an empirical type I error rate of 5.029e-08 (95% CI: [4.05e-8, 6.01e-08]) (**Table R1**).

Table R1 Empirical Type I error rates across various significance thresholds under the null hypothesis.

Threshold	Empirical type I error rate	95% CI
**0.01**	9.998e-03	[9.993e-03, 1.000e-02]
**0.001**	9.996e-04	[9.982e-04, 1.001e-03]
**0.0001**	9.995e-05	[9.951e-05, 1.004e-04]
**1e-05**	1.002e-05	[9.881e-06, 1.016e-05]
**1e-06**	9.316e-07	[8.894e-07, 9.738e-07]
**5e-08**	5.029e-08	[4.048e-8, 6.010e-08]

<table>
<tr><td></td><td>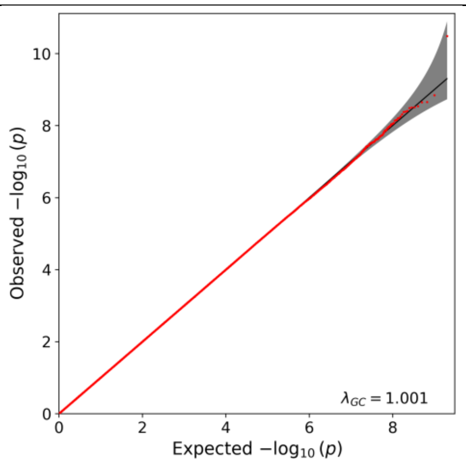

Fig. R1. Quantile–quantile (QQ) plot of null p-values from large-scale simulations. Under the null hypothesis of no genotype–phenotype association, the simulated p-values closely follow the expected uniform distribution, with the observed values largely tracking the diagonal expectation (red dashed line) and remaining within the 95% confidence band.

Together, these results provide direct evidence that JAGWAS produces well-calibrated multi-phenotype test p-values and appropriately controls Type I error across the full significance range, including at the genome-wide threshold.</td></tr>
<tr><td>Excerpt 1.3 (main text)</td><td>Not applicable.</td></tr>
</table>

REF2 – Point-to-Point response to REF2

REF2.1 – Predictive Reliability in Small-Sample Disease

Referee Comment	*UDIP-FA demonstrates predictive power for neurological and psychiatric disorders:*<br>*The authors added the sample size of each disorder and compared their model with a baseline model using standard measures from major white matter (WM) tracts. The authors report SCZ achieves the highest AUC despite having only 36 cases. This is paradoxical and suggests potential overfitting. While the authors argue in their rebuttal (REF1.2 – Prediction of UDIP-FA response) that this reflects "biological signal strength" rather than a statistical artifact, this interpretation is not adequately supported. With 128 features and only 36 cases, even with downsampling and cross-validation, the risk of spurious feature importance rankings is substantial.*<br>*Suggestion: Either (a) provide support to show that for small sample sizes the results reflect biological signal and not statistical artifact, (b) restrict analyses to disorders with adequate sample sizes (DEP, EPI, MS), or (c) clearly state in the main text that results for SCZ, AD, and PD are*

	exploratory and underpowered, moving detailed interpretation to supplementary materials.
Author Response	Thanks for your comments and suggestion. In the revised manuscript, we have followed your suggestion (c) and moderated our interpretation accordingly.<br><br>Specifically, we retained the overall predictive evaluation across all six disorders to preserve the completeness of the analysis, but restricted primary tract-level interpretation in the main text to the better-powered disorders (DEP, EPI, and MS，Fig.2d). Detailed feature-level results for smaller-sample disorders, including SCZ, AD, and PD, were moved to the Supplementary Information and are now explicitly described as exploratory and underpowered. We also removed the stronger interpretation that the high AUC observed in SCZ reflects biological signal strength, as we agree that this conclusion is not sufficiently supported by the current sample size.<br><br>In addition, we revised the Discussion to explicitly note that, in high-dimensional settings, limited case numbers may inflate the apparent variability of cross-validated classification performance and reduce the stability of feature-importance rankings. These revisions better align the strength of our claims with the statistical support provided by the available data.<br><br>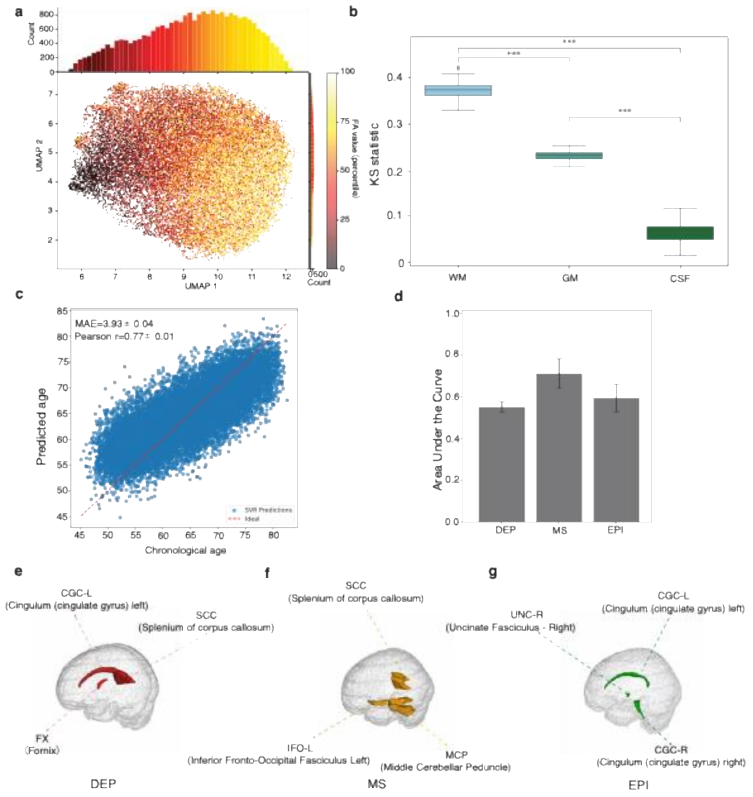

	**Figure 2: Characterization and clinical relevance of UDIP-FA features. (a**) 2D UMAP projection of UDIP-FA representations across all subjects, colored by mean FA values of Fornix.<br>**(b)** Kolmogorov–Smirnov (KS) statistics quantifying the distinctiveness of UDIP-FA features across three major tissue types: WM, gray matter (GM), and cerebrospinal fluid (CSF). WM regions exhibited significantly higher KS scores, indicating stronger discriminative patterns (***$P < 0.001$, Mann-Whitney U test). **(c)** Brain age prediction using UDIP-FA features. Scatter plot shows predicted versus chronological age, with a mean absolute error (MAE) of 3.93 years and Pearson correlation of $r = 0.77$. **(d)** Classification performance of UDIP-FA in distinguishing individuals with various brain disorders, including Depression (DEP), multiple sclerosis (MS), epilepsy and (EPI). **(e–g)** Brain regions identified as most predictive for DEP(e), MS (f), and EPI (g) using feature importance of top 3 features from the GBDT classification model.
Excerpt 2.1 (main text)	Line 145-151:<br>"Given the substantial differences in case numbers, primary tract-level interpretation in the main text focused on DEP, EPI, and MS, whereas results for AD, PD, and SCZ were treated as exploratory (Figure S9). The results demonstrated that UDIP has measurable predictive utility for DEP, EPI, and MS (Figure 2d), and offers complementary predictive value to traditional tract-based metrics in most disorders. Furthermore, we analyzed feature importance from the classification model to investigate potential disorder-related white matter patterns in the disorders retained for primary analysis"<br>Line 446-450:<br>"To minimize over-interpretation of potentially unstable feature-importance rankings in smaller case groups, we focused primary tract-level interpretation on disorders with relatively larger case numbers, while treating results from smaller-sample disorders (AD, SCZ, and PD) as exploratory and presenting them in the Supplementary Information (Figure S9)"

REF2.2 – Reliability and Stability of Feature Importance Interpretations

Referee Comment	*On a related point, in the same section, the feature Importance Interpretation from small samples needs some validity.*<br><br>*lines 162-165: "In AD, the critical UDIP-FA feature (dimension_7) was significantly enriched in the left cingulum (cingulate gyrus) (CGC-L), the genu of the corpus callosum (GCC), and the splenium of the corpus callosum (SCC), with a Bonferroni-corrected P < 0.05 based on permutation test results from PerDI."*<br>*These interpretations are derived from models trained on 63 AD cases. Feature importance rankings from gradient boosting with 128 input features and ~60 cases are highly unstable. The authors cite literature showing these tracts are "implicated in AD-related pathology," but this may reflect the model picking up on well-established group differences that would be detectable with conventional methods.*<br>*Suggestion: Add stability analyses (**e.g., bootstrap confidence intervals on feature importance rankings**) or acknowledge explicitly that these interpretations are hypothesis-generating only and require validation in larger samples*
Author Response	Thanks for your comments and suggestions. In response, we revised the manuscript in three ways. First, we removed the detailed AD feature-importance interpretation from the main Results and restricted primary tract-level interpretation in the main text to the better-powered disorders. Second, we moved the corresponding smaller-sample results, including AD, to the Supplementary files and now explicitly describe them as exploratory and hypothesis-generating. Third, we revised the Discussion to note that tract-level interpretations derived from feature importance may be less stable in disorders with limited case numbers and therefore require validation in larger samples.
Excerpt 2.2 (main text)	Line 150-157:<br><br>"Furthermore, we analyzed feature importance from the classification model to investigate potential disorder-related white matter patterns in the disorders retained for primary analysis. For MS, DEP, and EPI, the top-ranked UDIP-FA features showed biologically plausible tract-level enrichment patterns, supporting the utility of UDIP-FA for capturing disorder-relevant white matter variation (Figure 2e, f, g). In DEP, the important UDIP-FA feature (*dimension_37*) was significantly enriched in the left cingulum (cingulate gyrus) (CGC-L), and the Fornix (FX), with a Bonferroni-corrected *P* < 0.05 based on permutation test results from PerDI.

	These WM tracts have previously been implicated in DEP-related pathology [1, 2]"<br><br>Line 358-368：<br><br>" Importantly, although the model was not trained on disease labels, UDIP-FA features recapitulated associations with white matter tracts previously implicated in neurological disorders (e.g., the corpus callosum and cerebellar peduncle in MS) and showed significant correlations with deficits in information processing speed and motor coordination scores (e.g., SDMT and EDSS cerebellar subscores) [3, 4]， as well as established polygenic risk measures[5]. This observation is consistent with prior studies showing that unsupervised representations can reveal subtle pathology-related patterns that may be missed by aggregate measures. In addition, our analyses identified significant associations in disorders such as MS and ALS, further supporting UDIP-FA as a biologically informative and hypothesis-generating representation of white matter variation that may complement conventional phenotypes in neurodegenerative research. "

REF2.3 – External Validation and clinical utility of UDIP-FA

Referee Comment	*Major Comments:*<br><br>3) External Validation and clinical utility.<br>(Also raised in REF3.3 – External validation of UDIP-FA)<br><br>Lines 454-459 acknowledge this limitation:<br>"while our discovery-replication design confirmed internal reproducibility under standardized UK Biobank acquisition, we have not yet assessed the model's generalization to external cohorts with varying scanners or protocols"<br><br>For a method proposed as capturing "biologically and clinically informative brain phenotype" (Line 30-31), the absence of external validation is a limitation.<br><br>Suggestion: Please rephrase claims about clinical utility throughout. Specifically, revise the Abstract (Lines 30-33) and Discussion to position

	this as a proof-of-concept requiring external validation before clinical translation claims are warranted.
Author Response	Thanks for your comments and suggestions. To address it, we have revised the Abstract and Discussion to position UDIP-FA primarily as a biologically informative, proof-of-concept imaging phenotype rather than a clinically validated biomarker. We also clarified that the predictive analyses were intended to demonstrate biological informativeness rather than to establish a clinical diagnostic model, and we explicitly note in the limitation section that external validation will be required before claims regarding clinical utility are warranted.
Excerpt 2.3 (main text)	Line 25-27：<br>"Together, our results suggest that UDIP-FA provides a biologically meaningful view of white matter, complementing conventional ROI-based FA measures and offering a more refined way to study its genetic architecture."<br>Line 137-138:<br>"UDIP-FA captures biologically informative variation associated with age, sex, and brain disorders"<br>Line 345-348:<br>"Overall, UDIP-FA provides a biologically informative framework for advancing our understanding of WM biology and its links to brain health and disease, while further external validation will be necessary to determine its generalizability and clinical applicability"<br>Line 416-417：<br>"This perspective offers a possible roadmap for extracting biologically grounded candidate targets from broader statistical associations."<br>Line 450-454:<br>"Moreover, although our discovery-replication design confirmed internal reproducibility under the standardized UKB acquisition framework, we have not yet assessed the generalizability of the model in external cohorts acquired using different scanners or imaging protocols. Accordingly, further validation in independent datasets will be necessary before claims regarding clinical utility can be made."<br>Line 461-464:<br>"Importantly, UDIP-FA reveals previously unrecognized links between WM integrity and brain disorders, and highlights candidate therapeutic targets, supporting its value as a framework for generating biologically

	grounded hypotheses at the interface of imaging genetics and neuropsychiatric disease."

REF2.4 – Biological specificity vs. global brain morphology

Referee Comment	*Minor Comments:*<br><br>*1) Shared variance between UDIP-FA and global structural and anthropometric phenotypes.*<br>*The authors added Figure S4, showing the proportion of variance in the 128 UDIP-FA features explained by different sets of imaging-derived and anthropometric measures, estimated using canonical correlation analysis (CCA).*<br>*And added text on Lines 120-124, Lines 441-445, and Lines 715-717.*<br>*This explanation is plausible but raises the question on interpretation of "the biological specificity of UDIP-FA" (Lines 120-121). If UDIP-FA captures global brain development signals, is it truly providing WM-specific insights, or is it a general brain structure phenotype?*<br>*Suggestion: Add a sentence in the Discussion explicitly addressing whether the shared variance with cortical morphology is a limitation for WM-specific applications or an advantage for capturing integrated brain structure.*
Author Response	Thanks for your comments and suggestions. We agree that the shared variance between UDIP-FA and global structural phenotypes warrants clarification with respect to the biological interpretation of these representations. In the revised Discussion, we now explicitly note that the overlap of UDIP-FA with T1/T2-related or cortical structural signals should not be interpreted as contradicting its white matter relevance. Rather, this pattern may reflect coordinated brain-wide developmental programs and genetic pleiotropy, whereby structural and microstructural traits naturally co-vary across individuals[6, 7]. At the same time, we also acknowledge that this shared variance argues against an overly strict WM-only interpretation.<br><br>Importantly, our phenotypic analyses show that UDIP-FA explains substantially more variance in tract-level white matter metrics than in cortical morphology or anthropometric traits, supporting the interpretation that these representations remain preferentially anchored to white matter microstructure rather than serving as a nonspecific marker of global brain structure. We have revised the Discussion accordingly.

Excerpt 2.4 (main text)	Line 430-437:<br>" While UDIP-FA shares genetic loci with T1 and T2 modalities, this pattern likely reflects the extensive genetic pleiotropy underlying global brain development, in which structural and microstructural traits co-vary across individuals rather than representing isolated modality-specific processes[6]. Consistent with this result, prior work has shown that cortical morphology and corresponding white matter microstructure can undergo coordinated developmental change[7]. At the same time, our phenotypic analyses demonstrate that UDIP-FA remains most strongly anchored to white matter microstructure, supporting a modality-specific sensitivity rather than a nonspecific marker of global brain structure[8]."

REF2.5 – Code

Referee Comment	The code appears to be comprehensive and easy to follow, covering most aspects of the analysis, but a detailed assessment may be needed.
Author Response	We thank the reviewer for this comment. We are encouraged that the code was found to be comprehensive and easy to follow. To further strengthen the rigor of the analysis, we carefully re-examined the full pipeline and clarified key implementation details in the code documentation. We also performed additional checks to ensure the correctness and consistency of the analytical procedures. These revisions were made to improve transparency, reproducibility, and the overall robustness of the study.
Excerpt 2.5 (main text)	Not applicable

Reference

[1] Liao Y, Huang X, Wu Q, Yang C, Kuang W, Du M, et al. Is depression a disconnection syndrome? Meta-analysis of diffusion tensor imaging studies in patients with MDD. J Psychiatry Neurosci 2013;38:49-56.

[2] Hoogenboom WS, Perlis RH, Smoller JW, Zeng-Treitler Q, Gainer VS, Murphy SN, et al. Limbic system white matter microstructure and long-term treatment outcome in major depressive disorder: a diffusion tensor imaging study using legacy data. World J Biol Psychiatry 2014;15:122-34.

[3] Govindarajan ST, Liu Y, Parra Corral MA, Bangiyev L, Krupp L, Charvet L, et al. White matter correlates of slowed information processing speed in unimpaired multiple sclerosis patients with young age onset. Brain Imaging Behav 2021;15:1460-8.
[4] Hannoun S, Kocevar G, Durand-Dubief F, Stamile C, Naji A, Cotton F, et al. Evidence of axonal damage in cerebellar peduncles without T2-lesions in multiple sclerosis. Eur J Radiol 2018;108:114-9.
[5] de Mol CL, Jansen PR, Muetzel RL, Knol MJ, Adams HH, Jaddoe VW, et al. Polygenic Multiple Sclerosis Risk and Population-Based Childhood Brain Imaging. Ann Neurol 2020;87:774-87.
[6] Alexander-Bloch A, Giedd JN, Bullmore E. Imaging structural co-variance between human brain regions. Nat Rev Neurosci 2013;14:322-36.
[7] Jeon T, Mishra V, Ouyang M, Chen M, Huang H. Synchronous Changes of Cortical Thickness and Corresponding White Matter Microstructure During Brain Development Accessed by Diffusion MRI Tractography from Parcellated Cortex. Front Neuroanat 2015;9:158.
[8] Lebel C, Deoni S. The development of brain white matter microstructure. Neuroimage 2018;182:207-18.
